# International Guidelines for Photosensitive Epilepsy: Gap Analysis and Recommendations

**DOI:** 10.1145/3694790

**Published:** 2024

**Authors:** J. BERN JORDAN, GREGG C. VANDERHEIDEN

**Affiliations:** University of Maryland, College Park, Maryland, USA

**Keywords:** flash, flicker, games, seizure, standards, television, video

## Abstract

People with photosensitive epilepsy may have seizures caused by flashing lights, patterns, and video sequences. Because of this, there is increasing interest among researchers, developers, and companies toward making content safer. There are five major guidelines (from the U.K., Japan, ISO, ITU, and W3C) to limit risk in different technology domains that have been created since the mid-1990s. All address similar risk factors, but they are not fully harmonized and can be confusing. Furthermore, there have been significant changes in technology since the guidelines were introduced. This article compares and clarifies the guidelines, describes risk factors that have changed (such as the reduction of risk due to display technology), gaps in our knowledge, the coverage of new technology, and new characteristics (such as the duration and synchronicity of individual flash transitions) that may need to be accounted for. The authors suggest working values for new thresholds and propose updated guidelines.

## 1 Introduction

People with **photosensitive epilepsy (PSE)** may have seizures when viewing flashing lights; bold, regular patterns; or certain video sequences on televisions, computers, and mobile devices. Today, with an increasing number of screens and a wide variety of video content available from many sources, it is becoming increasingly likely that people with PSE (estimated at 1 in 4,000 of the general population [32]) will encounter content that is hazardous to them. While there are some strategies an individual can use to limit risk (avoiding known hazardous content, covering one eye with a hand, viewing content at a distance in a bright environment, etc.), many of these strategies only work for people who know about their own photosensitivity and the specific hazard ahead of time. It is uncommon to regularly screen for photosensitivity. Thus, there is an unknown population of people at risk who have not yet had a seizure or PSE diagnosis. This latent population was dramatically brought to attention when flashing sequences in a 1997 broadcast of a cartoon in Japan resulted in 685 children going to hospitals [41, 54]. To help protect all people at risk of PSE, and particularly the latent, at-risk population, PSE guidelines and standards were created.

Research into the stimuli that trigger such reflex seizures was incorporated into guidelines and standards starting in the 1990s [39]. Most early guidelines were intended for broadcasters, advertisers, and broadcast content creators to use in order to limit the number of viewers who might have seizures when viewing content on television. Early photosensitivity guidelines were also incorporated in consumer product accessibility guidelines [99]. In Japan and the U.K., where there was enforcement of PSE guidelines for broadcast television, there was some success in reducing the number of complaints and cases [94]. The guidelines aim to provide an acceptable level of protection while being relatively easy to implement [14].

Guidelines on limiting PSE risks can be used as the basis for analysis and screening tools. These tools can be useful at several levels: for content creators, for platforms and broadcasters who distribute content, for consumers who view content [84], and for auditors. Content creators can check their own videos for potential hazards. Organizations such as broadcasters, social media platforms, and media streaming services could screen videos before they are shown or otherwise made publicly available. Consumers could potentially use such tools to identify hazardous content before or during viewing to avoid or actively mitigate potential risk factors. Finally, auditors use guidelines and tools to check content for compliance for quality control or after a complaint.

Today, there are five major sets of guidelines and standards for limiting the risk of PSE. These apply to broadcasts [50, 55, 69], image and video content more generally [49], and web content [106] plus software and electronic documents through legislation of various countries. While there are many similarities between the guidelines, they are not identical. These technical guidelines can also be difficult to understand to developers and other groups that need guidance. Additionally, technology has continued to advance while much of the guidance has remained the same since the mid-1990s, when **cathode ray tube (CRT)** screens were commonplace. With newer technologies, faster frame rates, brighter screens, and a wider variety of devices with screens, changes or additions to the guidance may be appropriate.

Image and video safety is an important consideration in human–computer interaction, especially as larger and more immersive displays, games, videos, and other content are being developed. In this article, we review the current PSE standards and relevant medical literature and add three contributions:

- Compares the five international PSE safety standards (the first published in-depth comparison to do so), showing where they are harmonized and how they differ.
- Identifies new potential gaps and ambiguities in the guidelines by reviewing the changes in technology (including display technology, frame rates, dynamic range, and color spaces) since the thresholds were set.
- Addresses these gaps with recommend changes and additional thresholds to be considered for incorporation into PSE standards and future tools.

## 2 PSE and Danger from Screens

PSE is a *reflex epilepsy*, meaning that seizures can be reliably triggered by specific stimuli, such as flashing lights, patterns, or particular changes in color [32]. A diagnosis of photosensitivity is made by measuring brain activity with an electroencephalogram while flashing lights at different rates using **intermittent photic stimulation (IPS)** [57]. A person is diagnosed as photosensitive if their brain activity shows a **photoparoxysomal response (PPR)** [73]. It is possible for people to be photosensitive and have never encountered a stimulus that caused a seizure. Estimates for the number of people in the overall population who might exhibit PPR vary widely from 0.3% to 8% [78], but not all of these people have PSE. An oft-cited figure is that 1 in 4,000 people have PSE [32]. Photosensitivity is 1.5 to 2 times more common in females than males [101]. Photosensitivity is most common between the ages of 8–25, with a mean age of onset of 14 years and declining incidence after 20 years [81]. Most people do not outgrow photosensitivity, with one study showing 70% of people remained photosensitive after long-term follow up [38].

When somebody is photosensitive, their brain is hyperexcitable. Firstly, to precipitate a seizure, a critical population of neurons in the visual cortex of the brain must be stimulated [111]. The area of brain involved corresponds with the area of the visual field that is being stimulated [27]; thus, larger areas of a screen activate larger areas of the brain. Secondly, the activity of the neurons must be sufficiently rhythmic and synchronized [32, 111]. This synchronization in the neurons is set up by regular flashing, flicker, oscillation, vibration, phase reversal, or bold static patterns. In people with PSE, the compensatory mechanisms that prevent large-scale neural synchronization may be impaired [13, 100].

Television and video games have long been known to provoke seizures [42, 83]. Some of this was due to the inherent flicker of CRT television screens, but it is also due to hazardous content. In the U.K., a Golden Wonder Pot Noodle advertisement shown in 1993 caused seizures in at least three people [39]. This event led to the creation of PSE guidelines in the U.K. [39]. In 1997, a prime time showing of an animated cartoon (*Pocket Monsters* or *Pokémon*) resulted in hospital visits for 685 children [54] and more sought medical attention after the clip was shown in media reports later that evening. An official report concluded that over 10% of all viewers had reported symptoms, including headache and dizziness [92]. A sequence of 12 Hz alternation between large red and blue fields, with a relatively small 25-cd/m^2^ difference in luminance between states, was found to be the trigger [41, 54, 92]. Guidelines were subsequently updated to include red flashes as a risk factor [43]. Where PSE guidelines are enforced, there have been occasional advertisements and video sequences that have caused complaints since then. In a review of decisions published in broadcast bulletins from 2005 to 2021, we found that Ofcom (which regulates broadcast and on-demand television in the U.K.) found 30 instances of television broadcasts that exceeded their PSE guidelines, and 80% of the initiating complaints were submitted by viewers. A notable 2007 promotional video for the London 2012 Olympic games generated 30 complaints of PSE [40].

The internet and social media platforms are another source of potentially hazardous content. Users can easily post videos and animations, which may be hazardous. In what has been called one of the first physical attacks over the internet, malicious users posted flashing animations to online forums hosted by the Epilepsy Foundation in 2008 [76]. Since that time other attacks have occurred. Attacks with flashing animations used Twitter handles and hashtags associated with the Epilepsy Foundation in 2019 [2]. The Epilepsy Society was targeted in coordinated attacks using Twitter in 2020 [20]. Attacks have also been targeted at individuals—Eichenwald, a journalist, had a seizure when he opened a Twitter direct message with threatening text and a flashing image [56]. Simply looking away is not a reliable way for everyone to avoid hazards—with visual reaction times of 160 ms or more [4] a person may have already seen nine frames of a video at 60 fps before they can react.

Some platforms have implemented protective measures such as allowing users to turn off autoplay features and preventing searches for images using certain key terms [32, 88]. The TikTok platform has implemented automatic screening of user uploaded content and allows users to automatically skip content that is flagged as potentially dangerous [35]. The Zoom video conferencing system added a dimming function in 2022 to the application to limit PSE hazards in screen-share content [46]. Other online platforms have flagging and content moderation policies in place [34]. In 2023, Apple Inc. added settings in its mobile, computer, and television entertainment operating systems to allow users to Dim Flashing Lights in all displayed content [8]. In the UK, the Online Safety Act 2023 [115] includes language in section 183 making it a legal offence to intentionally send or post flashing content with an intent to harm a person with epilepsy (known as Zach’s Law [30]).

## 3 Current Standards and Guidelines

In 2004, the Epilepsy Foundation of America convened a workshop of experts to come up with a consensus on characteristics and thresholds to decrease the risk of PSE seizures. Their recommended thresholds for flashing and static patterns, styled as a “draft consensus,” were published in 2005 [44]. Wilkins, Emmett, and Harding proposed slightly updated recommendations later that year for regular patterns [109]. While these specific guidelines are not meant for conformance claims, the international guidelines are generally aligned with these expert recommendations.

For broadcast video media, there are three current standards that apply. The International Telecommunications Union is a specialized agency of the United Nations and has published ITU-R BT.1702 guidance with the most recent version from 2023 [50]. The **Office of Communications (Ofcom)** is the government-approved authority in the United Kingdom concerning broadcasting, telecommunications, and postal industries. Ofcom has published guidance about flashing and patterns that have the potential of causing PSE seizures [69] and has a broadcast code that requires compliance unless there are justifiable editorial reasons, in which case a verbal warning must precede the content [70]. Ofcom succeeded the U.K. **Independent Television Commission (ITC)** which had published earlier guidance [47] on PSE that is still occasionally referenced. Finally, specific to broadcasts in Japan, the Japan Broadcasting Corporation **(Nippon Hoso Kyokai (NHK))** and **Japan Commercial Broadcasting Association (JBA)** have published joint guidance, last updated in 2020 [55].

For image content (including video) on display screens, the International Organization for Standardization published the standard ISO 9241–391 [49].

Scoped to web content, the **World Wide Web Consortium (W3C)** has published the Web Content Accessibility Guidelines 2.x series of recommendations [102, 105, 106]. All three versions of the WCAG 2.x series remain simultaneously active as standards. These industry standards contain two relevant PSE **success criteria (SC)** for web content (SC 2.3.1 and 2.3.2). The latter, more conservative SC simply prohibits any flashing that occurs more than three times per second, while SC 2.3.1 contains thresholds on luminance, color, flash rate, and area akin to the other standards and guidelines mentioned above. The initial WCAG 2.0 PSE thresholds were set in consultation with Dr. Harding and Cambridge Research Systems, Ltd. The guidance on red flash thresholds was updated in 2023 in both WCAG 2.2 and the updated version of WCAG 2.1 to harmonize with those of ISO 9241–391, with input from the authors. Going forward in this document, we will only be considering the characteristics defined in SC 2.3.1 of WCAG 2.x rather than the rate-only limitation of SC 2.3.2.

SC from WCAG have subsequently been incorporated in accessibility regulations in many countries. In the United States and the European Union (through Section 508 of the Rehabilitation Act and EN 301 549, respectively), the WCAG guidelines, including the PSE SC, have also been applied to non-web digital documents and software that are purchased, procured, or created by governments. WCAG 2.0 was also adopted as ISO/IEC 40500:2012 because it may be easier for some countries or organizations to reference an ISO standard than an industry recommendation.

## 4 Available PSE Tools

Before automated analysis tools were available, manual analysis of video was time consuming. A one-minute section of potentially hazardous content might take a human observer 30–60 minutes to manually analyze frame by frame [40].

The Harding Flash and Pattern Analyzer (HardingFPA) is a commercial analysis tool for broadcast and video game content that has been available since 2001 [11]. Analysis machines can be purchased or rented, or analyses can be done on a per-video basis. Some commercial video quality control tools license the HardingFPA engine while other commercial tools, such as Vidcheck, Baton, Cel-Soft, and EditShare have their own PSE analyses.

Another well-known analysis tool is the **Photosensitive Epilepsy Analysis Tool (PEAT)** from the Trace R & D Center at the University of Maryland. The currently available version of PEAT is not fully open source, but it is free to use for web content. It uses an earlier version of the HardingFPA analysis engine, licensed from Cambridge Research Systems, that was adapted to meet WCAG 2.0 success criterion [21].

There have been several research tools that have been described in the last decade. Many of these tools have not been released for public use. Carreira et al. [23] used ITU-R BT.1702 as the basis for their analysis tool. They described algorithms and reported real-time analysis of sample videos and constructed video content.

Alzubaidi, Otoom, and Al-Tamimi [3] created a real-time system grounded in WCAG standards that could analyze video content when an HDMI cable was plugged in. Their system used a pipelined and parallel scheme using multiple processor cores to improve performance, and it outperformed PEAT and a serial-processing method they also developed. One caveat is that their system only has a frame buffer of 8 frames, so it may not catch some irregularly-timed flashing patterns that would not comply with WCAG.

Instead of taking a rule-based approach, Barbu, Banda, and Katz [10] created a system that used deep learning to detect hazardous video content and through video-to-video conversion to change it to be less hazardous. To train the model, they added potentially hazardous flashing to videos rather than using videos that were known to be hazardous. The model could filter some effects of flashing from real-world video samples. The authors had untrained observers rate flashing videos and did not compare their machine learning system output to available PSE guidelines.

South, Saffo, and Borkin [84] created PhotosensitivityPal to analyze both flashing and pattern hazards in short animations. Its rules are based on a combination of WCAG SC for flashing and guidance from [109] for patterns. However, in its current version, its area threshold uses a 25%-of-the-screen criterion instead of the more conservative area threshold in WCAG (see the later section on “Area of Flash” for a discussion of area thresholds.) PhotosensitivityPal is available as a prototype Chrome browser extension.

Kothari and Srivastava [59] describe a web app and Chrome browser extension called Flikcer based on ITU-R and Ofcom guidelines. Flikcer used machine learning techniques and could also remove frames from videos to provide a safer video download for people with PSE.

Tripathi et al. [97] created a browser extension to analyze YouTube videos and provide users advanced warnings of potentially hazardous content. The authors describe an algorithm that works in real-time to detect luminance flashes but did not mention minimum hazardous area thresholds.

In the past few years, there have also been several efforts from developers and others for PSE analysis. In 2019, a photosensitivity filter by Panteleev was added to the FFmpeg open-source project [74]. Its algorithm does not follow any specific PSE guidelines, but was created in consultation with people who have PSE. It was created to run efficiently and to filter or change the content on the fly to reduce the risk of flashing (personal communication). Another developer, West, spent several months of effort reading PSE guidelines and creating PSE analysis tools [107] as a browser plugin and as a web bot, although they are not currently being maintained (personal communication). In 2023, two large technology companies released open-source software for video content safety: Apple, Inc. released the Video Flashing Reduction tool [6, 7] and Electronic Arts, Inc. released the IRIS tool [28, 29]. Neither of these open-source tools have algorithms that strictly follow any of the international standards. The authors of this article have also addressed questions and consulted with other students and groups interested in tools and measurements on this topic.

## 5 Characteristics of Potentially Hazardous Flashing Content from the Guidelines

The goal of these standards and guidelines is to eliminate most hazardous situations, but it is impossible to eliminate all hazards for all people because of individual variation. While rare, some people have been reported to have a response to a single bright flash [44] or flashes from a front facing smart phone camera [19]. There has also been a report of someone who could exhibit a response due to mental imagery of flashing [5].

Across the standards and guidelines, there are several characteristics of flashes recognized as contributing to PSE seizures: (1) the magnitude of a flash, (2) the colors associated with states of a red flash, (3) the number of flashes within a particular time frame, and (4) the area of a flash. Three thresholds (either magnitude or color plus flash count plus area thresholds) must be met simultaneously for a sequence of flashes to be considered potentially hazardous. Thus, it is not considered hazardous if the flashing area is too small, the flashing is dim, or the flashing is too slow. A summary of the thresholds from the various guidelines is shown in Table 1.

The thresholds for each dimension of risk are a tradeoff between protecting a large population while still making the guidelines relatively easy to understand and allowing flexibility in video content. Thresholds in early guidelines, such as the one by ITC, were set using data from prior experiments. In general, these experiments tested one dimension at a time with the other dimensions set to highly stimulating values, so there is limited experimental understanding of the correlations and tradeoffs between dimensions. Using the 2001 ITC guidelines and available data, Binnie et al. modeled the risks assuming the dimensions were independent [14]. They estimated that of people who would likely exhibit a response to maximally stimulating flicker on a 60-inch television, only 0.1% would remain at risk if content conformed to the 2001 guidelines. From their model [14], it appears that adhering to the *flash rate* threshold is most protective for the most people, followed by adhering to the *luminance* change threshold, and then the *area* threshold.

### 5.1 Magnitude of a Luminance Flash

Luminance flashes are a change in luminance between dark and light states. The thresholds were set based on a combination of results of experiments that used static and phase shifting patterns with varying levels of luminance between light and dark stripes [14]. The thresholds are not based on flashes from photostimulators used for diagnosis of photosensitivity, since their flashes are bright: ranging from 400 to 10,000 cd/m^2^ or more [40, 42]. To simplify the thresholds, linear steps in luminance were chosen [14].

**Fig. 1.**
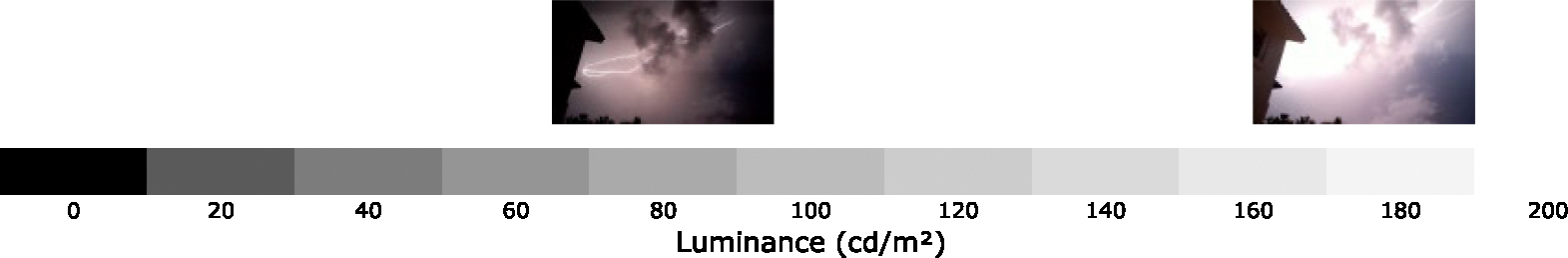
In most guidelines, luminance changes of 20 cd/m^2^ or more are potentially hazardous (corresponding to the grayscale blocks on the axis). Also shown are two frames of a flash of lightning that differ more than 100 cd/m^2^ on average. Stills from video by The Element [Pexels reuse license], via Pexels (https://www.pexels.com/video/thunder-and-flash-of-lightning-2657691/ ).

With **standard dynamic range (SDR)** content, the thresholds given in most guidelines are similar. General flashes, where the states differ more than 20 cd/m^2^, are potentially hazardous if the darker image is under 160 cd/m^2^ (see (Figure 1). The WCAG criterion uses a different metric (differences of 0.1 units of relative luminance when the darker image is under 0.8 relative luminance), but this converts to the same thresholds in cd/m^2^ given a 200 cd/m^2^ maximum screen luminance. Of people who would be sensitive to maximal black-white flashing it is estimated that 7% of them might still have a risk of PPR with flashing of 20 cd/m^2^ [14].

The Japan guidelines are not fully clear on the units of brightness used for SDR content, specifying differences in percent brightness. In an article comparing U.K. and Japan guidelines, it was reported that the analysis is done in analog IRE units without gamma correction, thus, “the Japanese guidelines are more tolerant to flashes or patterns which have a high average intensity but low relative contrast and less tolerant to those with low intensity but high relative contrast” [45]. Flashes with a moderate difference in brightness of 10–20% are allowed at a modestly higher rate (5 flashes/s) for up to 2 s. Brightness changes over 20% have the same 3-flashes/s allowance as other guidelines.

Two standards, ITU-R BT.1702–2 and the Japan broadcast guidelines, address **high dynamic range (HDR)** content, where images can be brighter than with SDR content. In both, the critical differences in luminance are defined in cd/m^2^ units when the darker state is darker than 160 cd/m^2^. In ITU-R, up to a 20 cd/m^2^ difference in states is allowed in this darker range, which matches the respective SDR criteria. In the Japan guidelines, there are two ranges: no more than 5 flashes/s for up to 2 s when the difference is 20–40 cd/m^2^ and no more than 3 flashes/s when the difference is ≥40 cd/m^2^. When the darker state is above 160 cd/m^2^ with HDR content, both guidelines use luminance contrast criteria (rather than luminance difference). In the ITU-R standard, a potentially hazardous luminance transition is when the darker state of the transition is above 160 cd/m^2^ and when the Michelson contrast (Equation (1)) between the two states equals or exceeds 1/17 (where $L_{\mathrm{High}}$ is the luminance of the brighter state and $L_{\mathrm{Low}}$ is the luminance of the darker state)

(1) $$\frac{L_{\mathrm{High}}\;-\;L_{\mathrm{Low}}}{L_{\mathrm{High}}\;+\;L_{\mathrm{Low}}}.$$

The Japan HDR standard has two ranges for flashes when the dark state is above 160 cd/m^2^. Where the brightness change is moderate, between 1/8 and 1/4 of the darker state (Michelson contrast of 1/17–1/9), then flashing must not exceed five times per second, and that rate is limited to 2 s of duration. When brightness changes exceed 1/4 of the darker state (Michelson contrast *>*1/9), then such flashing cannot occur more than three times in any second.

### 5.2 Magnitude of a Red Flash

Flashes that involve a deep, long-wavelength red color are potentially dangerous [89, 91], even if there is little luminance difference between the two states. Long-wavelength red colors excite only the long-wavelength sensitive cones of the eye without producing inhibitory effects from short- or medium-wavelength sensitive cones [15, 24, 41]. It is difficult to stimulate only short- or only medium-wavelength cones because of the overlap of cone sensitivities [15]. This leads to increased risks of a photosensitive response for long-wavelength red colors.

**Fig. 2.**
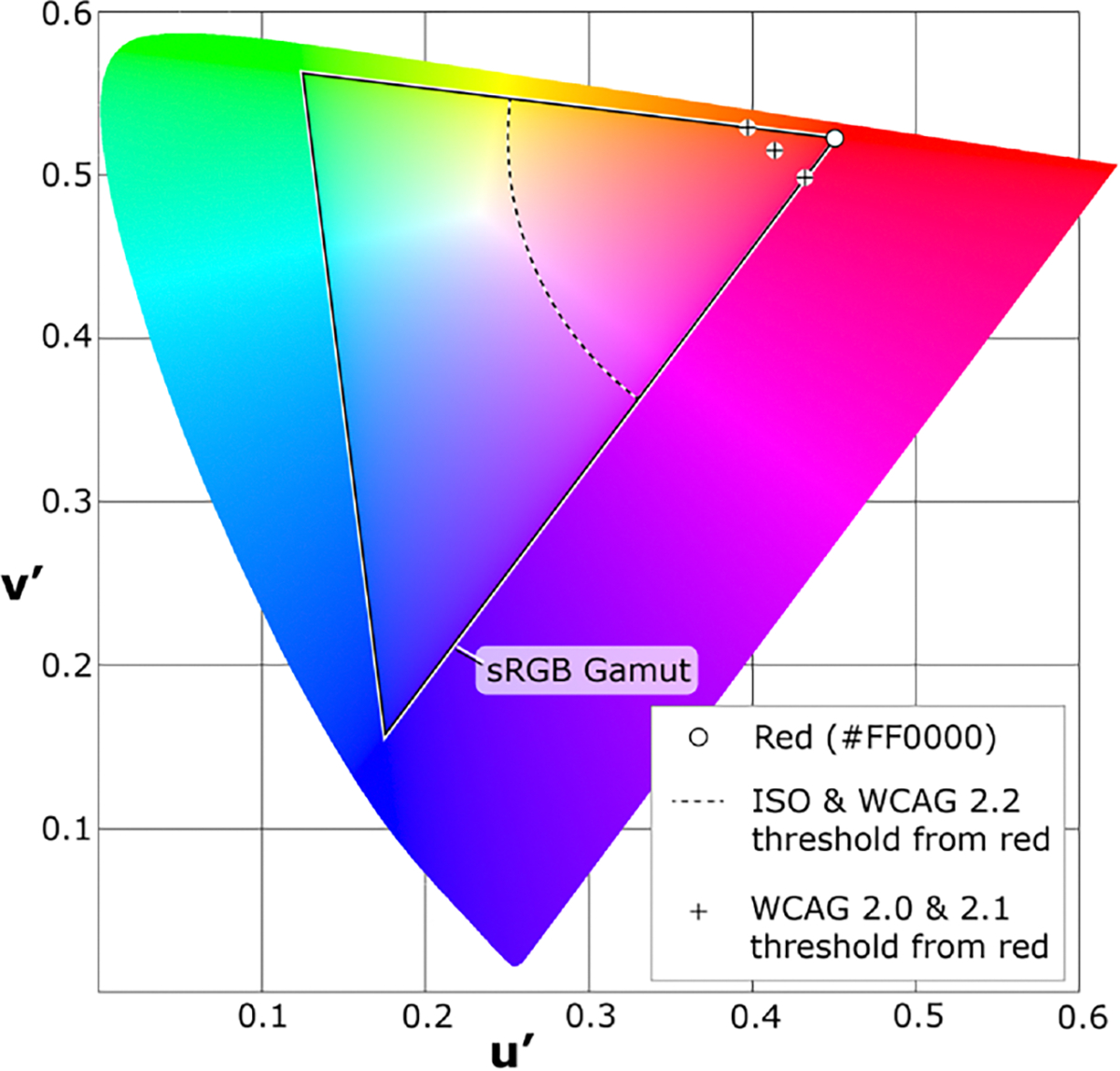
The CIE 1976 UCS chromaticity diagram showing the sRGB gamut (large triangle) and values of select red colors (marked with plus “+”) that differ enough in WCAG 2.0 critical values from pure red (#FF0000, in the upper right, red corner of the gamut triangle) to be considered a potentially hazardous transition. The harmonized ISO and WCAG 2.2 critical difference is represented by the dotted arc. Picture adapted from original by Adoniscik [Public domain], via Wikimedia Commons (https://commons.wikimedia.org/wiki/File:CIE_1976_UCS.png ).

All the current guidelines mention that red flashes are a potential problem, but most do not give specific thresholds. Only two guidelines, ISO 9241–391 and WCAG 2.×, define colors considered as “saturated red” and the minimum color difference between two states of a potentially hazardous red flash (see Figure 2). The WCAG definitions are styled as a working definition in a note and are thus non-normative.

The saturated red definition is the same in ISO and WCAG (Equation (2)), where R,G, and B are the normalized, linear, gamma-expanded values of the red, green, and blue channels (each varying between 0 and 1, inclusive)

(2) R/(R+G+B)≥0.8.

The actual colors considered to be saturated red depends on the color space and display technology. For example, the saturated red area of the analog **phase alternating line (PAL)** television standard includes some reds that are not as deep as the saturated reds of the analog NTSC standard [49].

The ISO and WCAG 2.2 standards differ from versions 2.0 and 2.1 (as first published in 2018 [103]) of WCAG in the color threshold difference between two states of a red flash. The new WCAG 2.2 and the 2023 update of WCAG 2.1 are harmonized with ISO, where the two colors forming a transition must differ more than 0.2 units on the CIE 1976 UCS chromaticity diagram (which was constructed so that spatial distance on the UCS diagram is relatively consistent to the human perception of differences between colors). In the earlier WCAG 2.0 and WCAG 2.1 as published in 2018 [103], a critical value is calculated for each state using Equation (3). The two colors forming a transition must have critical values that differ more than 20 units.


(3) $$WCAG\;2.0\;critical\;value=\left\{\begin{array}{ll} (R-G-B)\times 320 & \mathrm{if}\;R-G-B>0\\ 0 & \mathrm{otherwise} \end{array}\right..$$

### 5.3 Rate of Flashing (Luminance and Red Flashes)

**Fig. 3.**
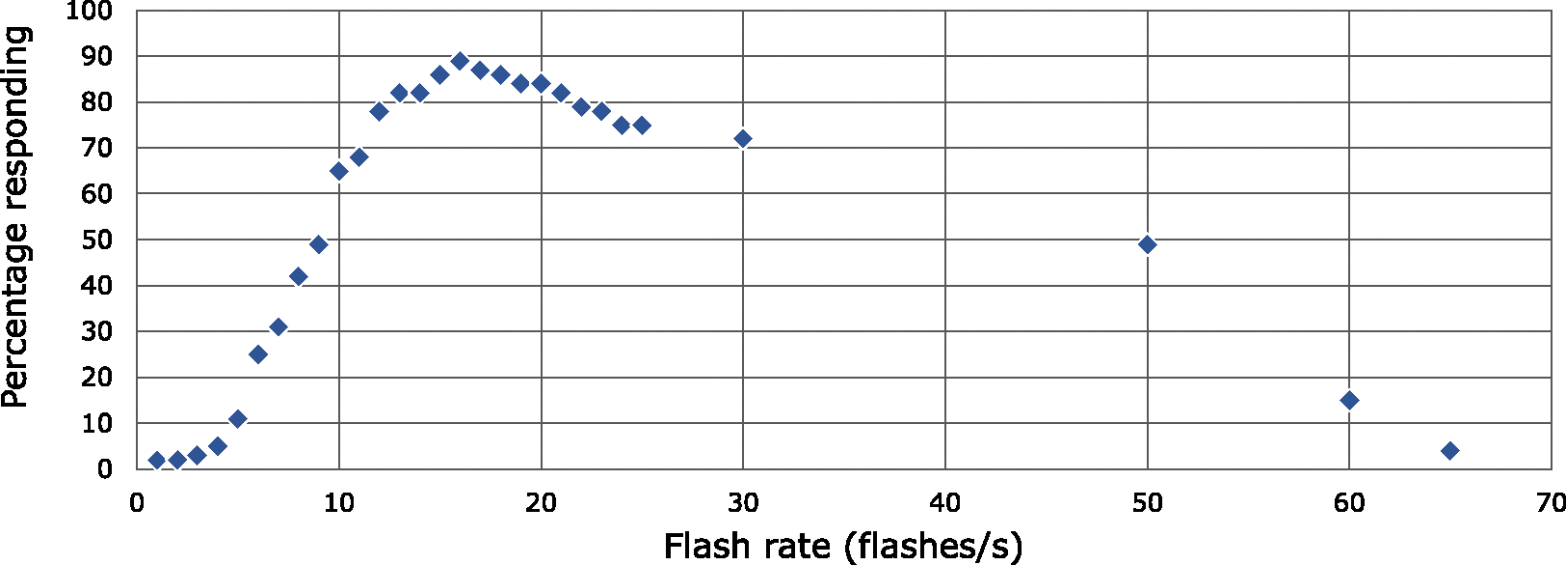
The percentage of people (*n* = 170) with PSE responding with PPRs at different flash rates. Only 3% had PPRs at 3 Hz. The response rate quickly goes up to nearly 90% responding at peak of 16 Hz followed by a decrease in people responding as the frequency increases beyond the peak. At 65 Hz, only 4% of people responded. Data from [42].

A flash is a dark-light or light-dark transition pair for luminance flashes and to-away or away-to transition pair with saturated red colors for red flashes. The frequency of a series of flashes has an effect on the number of people who might exhibit a response (see Figure 3).

All the guidelines, except the Japan guidelines, have the same lower limit for flashes: there can be no more than 3 flashes within any 1-s period. Thus, a sequence of seven alternating transitions within a second (i.e., 3.5 flashes) would potentially constitute a failure.

For Ofcom and ITU-R, there is a qualification on the spacing of flashes that is not present in the others. In the ITU-R and Ofcom standards it is acceptable if the leading edges of two successive flashes are separated 360 ms or more in a 50 Hz environment (i.e., 9 frames or more at 25 frames per second [fps]) or 334 ms in a 60 Hz environment.

The Japan broadcast guidelines are more complex when it comes to the rate of flashing because the allowed rate depends on the screen area and the difference in brightness.

- When a transition is classified as an *inversion* or *scene change*, then only up to three such transitions are allowed per second. In practice when using the HardingFPA analysis tool, this is when 80% or more of the screen has a 20% or greater change in brightness [22].
- For moderate flashes with a 10–20% brightness change, there can be up to 5 flashes (10 alternating transitions) per second for up to a 2-s period.
- For differences in brightness greater than 20% (and between 25% and 80% of the screen area), there must be three or fewer flashes per second in concordance with the other guidelines.

For HDR content in Japan, there are analogous ranges with thresholds that have different units. The ISO standard is the only one with a maximum frequency, where all flashing is allowed if there are more than 65 flashes in a second. Today, there is practically no pre-recorded content that approaches this upper safe limit; common frame rates today are 24–60 fps for television and 24 fps for theatrical motion pictures. Note that games have higher frame rates that are ever increasing, and the content is generated rather than pre-recorded, so they pose additional testing challenges.

In three standards (ISO, Ofcom, and ITU-R), there is mention of cumulative effects of extended flashing that is just below thresholds. Those three guidelines acknowledge that a sequence of flashes over a time period of 5 s might be problematic even if under the thresholds. No mention is given in the guidelines about any lower thresholds of luminance, area, or frequency that might be used for analysis.

### 5.4 Area of Flash

The larger the area of flashing, the more of the visual cortex of the brain is involved, and the more likely it is to be hazardous [14]. The critical area of stimulation of the visual cortex varies by individual [110]. Area thresholds for the population were set based on experiments done with brightly-lit, high contrast, grating (striped) patterns [14, 110]. Since flashing and pattern photosensitivity have similar effects, it is expected that hazardous flashing areas are similar.

Patterns and flashing in the center of a person’s field of view are more provocative than patterns and flashing that are toward the periphery [42, 110]. There is a higher density of photoreceptors in the fovea (middle of the visual field) and fewer photoreceptors away from the center.

**Fig. 4.**
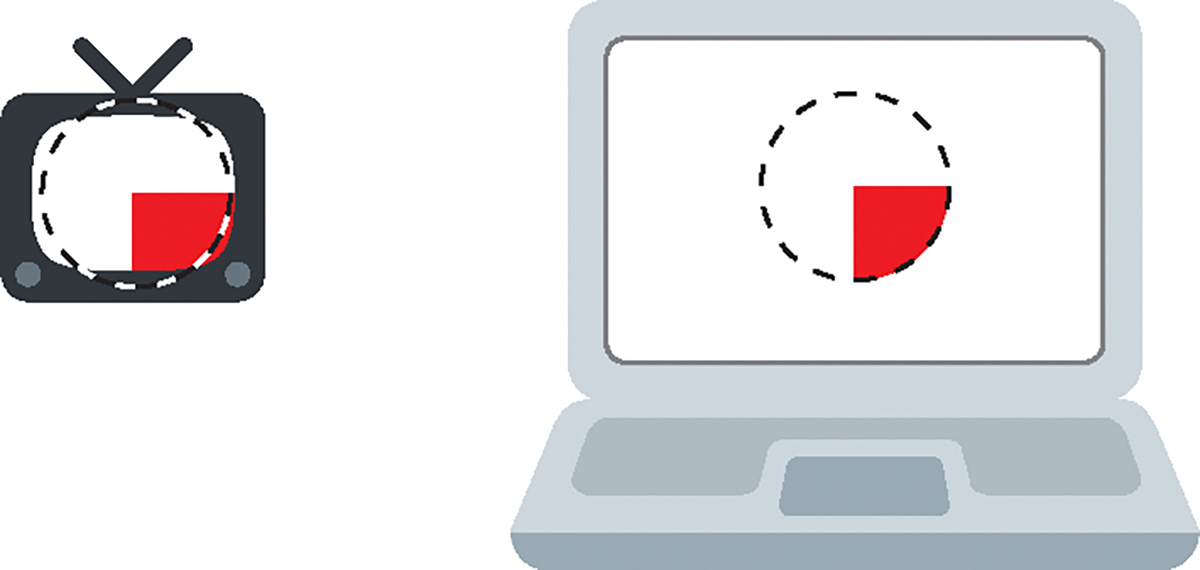
An illustration of the area threshold (shaded red) on televisions and computer displays, which are viewed at closer distances. The threshold area is 0.006 steradians, which is a quarter of the central 10° field of view (dashed circles). This is approximately 25% of a television screen at typical viewing distances. Note that flashing areas do not need to be contiguous, or the specific shape shown here. Device illustrations from Twitter Twemoji [CC-BY-4.0 license] (https://github.com/twitter/twemoji/ ).

A solid angle area threshold of 0.006 steradians is given in the expert consensus, but 25% of a television screen at a typical viewing distance was also given as a practical threshold [44]. (Note that steradians are a measure of solid angle, where a full sphere has a solid angle of 4*c* steradians.) All of the guidelines except WCAG require flashes be constrained to 25% of the screen (see Figure 4). This 25%-of-the-screen area threshold was estimated to remain a risk for 39% of people who would otherwise respond if they watched a full 60-inch TV flash at typical viewing distance, maximum brightness, and maximum rate [14]. The WCAG SC (again worked out in consultation with Dr. Harding and Cambridge Research Systems) is more complex: “the combined area of flashes occurring concurrently occupies no more than a total of 0.006 steradians within any 10° visual field on the screen (25% of any 10° visual field on the screen) at typical viewing distance.” [106] Put another way, the WCAG SC allows a maximum flashing area of one-quarter of any potential 10°-sized subset of the screen. If one assumes pixels as specified by the CSS 3 reference pixel [104], a circle with a diameter of 235 CSS px would be 0.006 steradian in solid angle and would meet the area threshold.

## 6 Gaps in the Guidelines

The PSE guidelines are similar to each other, but they are not fully harmonized. Furthermore, technology has progressed since the original research from which most thresholds in the various guidelines were set. Progress has reduced some risks and increased others. New technology has also revealed some gaps in the guidelines and in knowledge about PSE risk factors.

### 6.1 Screen Technology and Luminance

Displays have changed significantly since the standards were introduced. When the foundational research and subsequent standardization and threshold setting were taking place, CRT technology was the dominant screen technology for televisions and computer monitors. These analog displays have since given way to digital technology. These technology changes have mixed effects on the risks of a photosensitive response.

#### 6.1.1 Flicker Inherent to Display Technology.

When the PSE thresholds were set, televisions used CRTs. With CRT technology, images are presented by rapidly “scanning” the phosphor coating inside the screen with a swiftly-moving spot of electrons that would excite the phosphor to emit light. Thus, a bright dot of light would quickly scan across the screen line by line and the persistence of the human vision system would perceive a solid image. A dot of excited phosphor would rapidly decay in intensity until the next time it was scanned. As a result, at any given instant, the majority of the screen face was not illuminated. In addition, there was a blanking interval period where there was no phosphor excitation, between the last line at the bottom of the screen, and the next line at the top. A consequence of this scheme was an inherent flicker. The image frame rate was 25 Hz for PAL and SECAM (formats used in much of the world) and 29.97 Hz for NTSC standards (used in much of the Americas, Japan, and a few other countries). These frames were split into two interlaced half-resolution fields thus effectively doubling the refresh rate to 50 Hz (PAL and SECAM) or 59.94 Hz (NTSC). In Europe, with PAL and SECAM and a 50 Hz refresh rate, most children at that time had their first PSE seizure from watching TV [40]. Flicker was also more hazardous with close viewing or if the TV was out of adjustment [42]. Computer monitors using CRT technology typically had faster frame rates of 70 Hz or more. There were also high frame rate CRT televisions that were available, and a study showed that 100-Hz TVs evoked significantly fewer responses than 50-Hz TVs for people who were sensitive to patterns [33].

Today’s display technologies have different flicker characteristics than legacy analog displays, in many cases reducing or eliminating inherent hazardous flicker. Some displays also have different modes that might introduce flicker such as reduced brightness or with motion blur reduction features enabled. In all cases, higher refresh rates decrease both the hazard for PSE and the incidence of other adverse health effects such as eye strain, fatigue, and headaches [18, 58, 112]. The limit of human perception of flicker is the **critical flicker fusion frequency (CFF)** with typical rates of 50 Hz–90 Hz, but under certain circumstances people might perceive visual flicker at rates over 500 Hz [25]. Flashing faster than the CFF can still cause adverse effects even if it is unlikely to cause a seizure [62].

For dimming, many displays (especially plasma displays and many **organic light-emitting diode (OLED)** displays) employ **pulse-width modulation (PWM)**, which can introduce flicker that may become more apparent at lower brightness levels. With PWM, a backlight or the displayed image itself is made dimmer by flickering the backlight or image and reducing the amount of time that it is brightly displayed. For example, a screen turned down to 25% brightness with PWM would quickly flicker such that the whole screen (or scanning portions of the screen) would be on for 25% of the time and off 75% of the time. Flicker rates associated with PWM vary with some being very fast (*>*1000 Hz) and a few being as slow as 60 Hz.

There are flicker free ways of implementing dimming. With some technologies, dimming can be accomplished with through analog methods, such as reducing the applied voltage. This is used with the backlights of many **liquid-crystal display (LCD)** computer displays and TVs to eliminate flicker. Some other dimming schemes adjust the color transfer function of the display to skew images darker without introducing flicker. Some displays have refresh rates that are so fast that flicker is not perceptible. Some other displays implement hybrid schemes that involve combinations of approaches and only utilize PWM dimming at low brightness levels.

Some televisions and displays designed for gaming have modes to reduce the apparent blurring that can occur with fast onscreen motion. Many of these enhanced motion features introduce flicker through backlight strobing, scanning backlights, or black frame insertion [112, 114].

Active shutter **three-dimensional (3D)** systems can be used with projectors and other types of displays. This 3D technology incorporates glasses with digital shutters that quickly and alternately block each eye in sync with the right and left images that are displayed. Therefore, when the projector or display has a refresh rate of 120 Hz, each eye sees an independent image flashed at a rate of 60 Hz. Wearing active shutter glasses can cause severe flicker when switching to **two-dimensional (2D)** content or viewing other displays [77].

Legacy film projection in the cinema also had flicker due to the rotary shutter system. In the case of a physical strip of film, after a frame is projected the film needs to be physically pulled down to position the next frame. Unchecked, the pulldown motion would cause a vertical smearing to be projected, therefore a rotating shutter is employed to block the light from the projector while the film is being pulled down. This rotating shutter is split into three so that each frame is flashed onto the screen three times before the next frame is pulled down. This results in an effective flash rate on screen of 72 Hz. Today, most theaters worldwide are using digital projection systems. With some systems, such as a three-chip **digital light processing (DLP)** technology (using micro mirrors), the image is essentially continuous because of very high modulation rates.

Single chip DLP projectors for home use have color flicker artifacts. These projects use a rotating color wheel and project rapid, successive flashes of the red, green, and blue color channels. Through eye movement, these color flicker artifacts can be perceived, often referred to as the “rainbow effect” [64]. Some modern single chip DLP projectors use a 120 Hz or 240 Hz refresh rate in an effort to reduce this characteristic.

Another projection technology uses lasers to scan a dot of light across the screen, in a way that is similar to that of legacy CRTs as described above. These systems also pose similar flicker considerations, and here as well, the solution is to increase the scan or refresh rate.

The risks associated with more exotic displays depends on the display technology and their refresh rates. Most **virtual- and augmented-reality (VR and AR)** headsets, such as the HTC VIVE, Meta Quest, Microsoft HoloLens, and Apple Vision Pro—including small displays such as on the Google Glass Enterprise—use flat panel digital screen technologies with lenses, prisms, wave guides, or beam splitters. The hazard would be related to the flicker inherent to the underlying technology and its refresh rate. A multi-view display (where different images can be shown to different viewers looking at one display), the Parallel Reality displays by Misapplied Sciences Inc. [17], do not use scan lines and would look like displays with a 60 Hz refresh rate to users (personal communication), and thus is not likely to cause harmful flicker. However, there may be flicker inherent to other future technologies—for example there are some prototype virtual retinal displays that use lasers to “paint” images directly on the retina [1]. It remains to be seen what the refresh rate for these technologies will be and if these future displays have the same inherent flicker effect as scan lines of an electron beam creating images on a CRT TV.

#### 6.1.2 Changes in Color Rendering and Gamut.

**Fig. 5.**
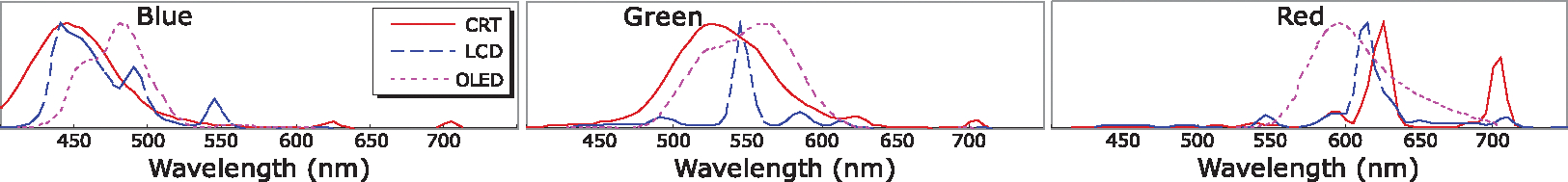
Spectra of the three primary colors (blue, green, and red) on three screens with different display technologies (CRT, LCD, and OLED). Most notable are the two red peaks of the CRT at 625 and 704 nm. Neither of the other screens output light with peak wavelengths that long. Figure adapted from [71] (used with permission).

Flashing that involves long-wavelength red light (wavelengths longer than 600 nm) has been found to be more likely to cause a response than white light or light of other colors, even at lower intensities [15, 41, 92, 95]. Color CRT displays typically emit more light of long-wavelength red than do current technologies. Long-wavelength red light was implicated in the infamous 1997 Pokémon episode [41, 92]. After the incident, researchers investigated televisions in the homes of 13 photosensitive children and found that those who had seizures viewing that episode had televisions with more luminance energy in the long-wavelength red part of the spectrum (defined for that study as between 720 and 780 nm) than the televisions of other photosensitive children who did not exhibit a response [93]. Note that colors in the 700+ nm range are where the response of human vision is low [87]. This elevated long-wavelength red output was present no matter what color was displayed on the screen and increased as television sets warmed up. They hypothesized that the heating of the shadow mask (a component of CRTs that focuses the screen into RGB subpixels) in some CRTs would emit long-wavelength red light and might predispose viewers to respond more readily to red flashing [93]. Adding to the risk associated with long-wavelength red light, the red color displayed on CRTs with the common P22 phosphor has a jagged spectrum with two sharp peaks at 625 and 704 nm (see Figure 5).

Modern color spaces do cover a wider gamut or range of colors than content in the past. This would include nominally deeper red hues than common, legacy color spaces such as sRGB. However, wide-gamut displays are unlikely to output red light with as long a wavelength as with CRTs. The red primary of the wide-gamut color space defined in both Rec. 2020 [51] and Rec. 2100 [52] has a wavelength of 630 nm. Even the ultra-wide gamut of the Kodak ProPhoto RGB (ROMM-RGB, specified in ANSI/I3A IT10.7666) synthetic working color space used for color grading work (which has imaginary B and G primaries) has a red primary of 700 nm. Both of these red primary wavelengths are shorter than the deep red 704 nm peak of CRT red phosphors and the other long-wavelength red light that CRTs produce.

In the visual discomfort literature, there is some evidence that the magnitude of the difference in chromaticity matters more than one color specifically being red. Greater chromaticity separation in striped (grating) patterns resulted in greater discomfort scores [37] and alpha suppression in the visual cortex for people who did not have PSE [36]. In Haigh, Cooper, and Wilkins [36], the authors replotted results from an earlier article by Parra et al. that had showed that flashing involving red was more hazardous to people with PSE than other colors [75]. When replotted against chromaticity separation, Haigh et al. found that the occurrence of PPRs in [75] was also correlated with chromaticity separation, which accounted for 75% of the variance [36]. This result suggests that further research may be needed to understand the effects of chromaticity separation and long-wavelength red on PSE and set better thresholds.

#### 6.1.3 Greater Screen Contrast Ratios.

Different screen technologies have different absolute contrast ratios between darkest and brightest content. LCD-type displays have a neutral-white backlight that shines through a liquid crystal layer that filters, colors, and reduces the light that is transmitted. LCD panels do not have completely dark blacks because some of the backlight illumination bleeds through. OLED displays have pixels that can be fully off, for higher contrast ratios compared to LCD displays. The higher maximum contrast ratios of some technologies do not impact the PSE calculations because black content calculates to 0 luminance regardless of the display. Technologies with better contrast can show subtle differences between black and very dark content, but this does not affect the photosensitivity risk.

#### 6.1.4 Brighter Displays.

Many displays available today can reach higher brightness than earlier analog counterparts. A person might set their display to a high luminance, which would cause other luminance levels to be scaled up. For example, what was a flash with a 20 cd/m^2^ difference (right at the threshold) on a display with a maximum of 200 cd/m^2^ might now be around 50 cd/m^2^ on a display with a 500 cd/m^2^ maximum.

Most of the guidelines (except WCAG and the 2005 expert consensus), specify a maximum display luminance of 200 cd/m^2^ for viewing SDR content (note that this is brighter than the 80 cd/m^2^ specified maximum in the sRGB color space [48]). The ITU-R and Japan guidelines go further and define HDR maximum luminance of 1,000 cd/m^2^ for **Hybrid Log-Gamma (HLG)** and 10,000 cd/m^2^ for **Perceptual Quantization (PQ)** content. While not explicitly stated, underlying the WCAG SC is a 200 cd/m^2^ maximum luminance assumption, such that 0.1 steps of relative luminance match the 20 cd/m^2^ steps of the other guidelines.

This 200 cd/m^2^ maximum luminance value holds reasonably well for many indoor viewing environments. In one study on television viewing in homes, the mean preferred peak viewing luminance was 160 cd/m^2^ for younger (mean age 22) and 248 cd/m^2^ for older participants with a mean age of 71 [65]. When producing HDR TV content, 203 cd/m^2^ is used as the white reference [53] with higher luminance reserved for reflections, shine, and specular highlights. Similarly, in indoor office environments, workers generally use their screens with luminance less than 200 cd/m^2^[72].

Of devices that people use every day, smartphones have the highest potential luminance and thus might pose a higher risk for PSE than under the guidelines’ assumed viewing conditions. Popular high-level smartphones of today have maximum typical brightness of 600–1,000 cd/m^2^ and peak HDR brightness of 1,200–1,800 cd/m^2.^ For battery savings, manufacturers recommend people use automatic brightness adjustment mode that adapts automatically for ambient lighting. Mobile phones are used in a wide variety of settings, but a study found that 80% of use is in indoor or nighttime environments with ambient light levels of 30 lux or less [80]. Even under high ambient illuminance levels of 5,000 lux (roughly the illuminance in the shade outside on a sunny day), college-aged participants generally preferred screen luminance levels under 200 cd/m^2^ [80].

Under bright ambient conditions, there are counteracting effects on photosensitivity risk. Two factors reduce the risk: (1) bright ambient lighting reduces the effective contrast of the display because of ambient light reflection and (2) the pupil of the eye contracts to let in less overall light. However, to improve legibility, people may increase the luminance of their display under such conditions. A brighter display increases the risk of a response because nominal 20 cd/m^2^ changes in brightness would actually have a greater magnitude. The exact relationship between these factors and PSE is not fully understood.

Even with changes in technology, the current luminance threshold steps are reasonable for analyzing the majority of content where the exact display characteristics are unknown. In most indoor viewing environments, many viewers likely to choose a maximum luminance around the 200 cd/m^2^ assumed by guidelines. Older people might turn up the brightness a bit further, but they are not a high-risk population for PSE. However, where specific display settings are known (e.g., a kiosk with known hardware or a future tool running in an operating system or in a processor on display hardware), then the luminance changes can be calculated with known pixel luminance values.

#### 6.1.5 Viewing in Dark Environments.

The human visual system has a wide dynamic range; able to see faint stars in the sky at night and scenes in the brightest midday sun. However, the visual system can only operate in a narrower range of luminance to which it is adapted, which takes time ranging from seconds to minutes [31]. This simultaneous dynamic range is about 3.7 log units [60] (e.g., 0.2–1,000 cd/m^2^).

Thus, with eyes adapted to dark ambient conditions, such as in a movie theater, the measured luminance of what is perceived as “very bright” is much lower than with typical indoor environments. Cinema has been known to be safer than home viewing because of relatively low luminance [42], but there have been cases of feature films in recent years that have caused seizures, resulting in warnings from the studio, and in some cases re-editing of the film to reduce the risk [32]. For cinema screens, the defined standard for peak white is 48 cd/m^2^ [26]. While this may seem low, the brightness perception is high due to the dark-adapted condition. None of the PSE standards are specifically for content in such dim viewing environments—they assume a brighter environment where a reference white of 200 cd/m^2^ is comfortable and reasonable. As such, it is important to recognize that darker environments may provoke a reaction with smaller changes in luminance. The actual level of protection is not known if current PSE guidelines were to simply be scaled down proportionally for dark-adapted viewing in a movie theater (i.e., scaling reference white from 200 to 48 cd/m^2^ and the luminance difference threshold from 20 to 4.8 cd/m^2^).

### 6.2 Screen Sizes and Viewing Distances

A flash or pattern that appears larger is more provocative, because more of the brain is involved [110]. The perceived size of a flash or pattern on a screen, depends on the size of the screen and the distance at which it is viewed. Today, televisions and computer monitors are available in a wider range of sizes than before. People are also using many other screens, including mobile phones, tablet computers, watches, and AR and VR headsets. All these screens are used at widely varying distances. If people are viewing screens so that they are much larger in their vision than the guidelines had anticipated there would be an increased risk of photosensitive responses.

The viewing of different devices can be compared using measures that are independent of physical distance, such as angle of view and solid angle. A *viewing angle* is the angle formed at the eye between opposite edges of a viewed object. For a variety of devices, Table 2 shows viewing angles and the percentage of the screen that forms a solid angle of 0.006 steradians, which is the hazardous area threshold [44]. The table also shows model-estimated percentages of people with PSE who would respond if only the middle 25% of the screen was flashing as calculated with the model in [14] and detailed in the Appendix.

#### 6.2.1 Comparing Area Thresholds.

Except WCAG, all the other guidelines set the area threshold at 25% of the screen. In the past, with typical TV viewing distances, 25% of the screen roughly corresponds to a solid angle of 0.006 steradians [44]. However, today, people often sit closer to their television screens and much closer to their computer displays (see Table 2). This results in the display taking up a wider angle of view and creating a greater risk of a response because 25% of the screen is a much wider solid angle than 0.006 steradians.

When describing pattern guidelines and rationale, Wilkins, Emmett, and Harding proposed limiting patterns to a combined solid angle of only 0.006 steradians if large, future screens were deemed to be problematic [109]. The WCAG approach based its area threshold for flashing on 0.006 steradians. However, instead of limiting flashing to a total solid angle area of 0.006 steradians, the limit is 0.006 steradians of any 10° subset of the screen (representing a person’s central vision where the risk is higher). There are more light sensing cells in the middle of ones’ vision, such that, “Once 10 degrees of the visual field is affected by flickering or patterned stimuli, further increases in area of the affected visual field has little significance for the photosensitive response” [44].

**Fig. 6.**
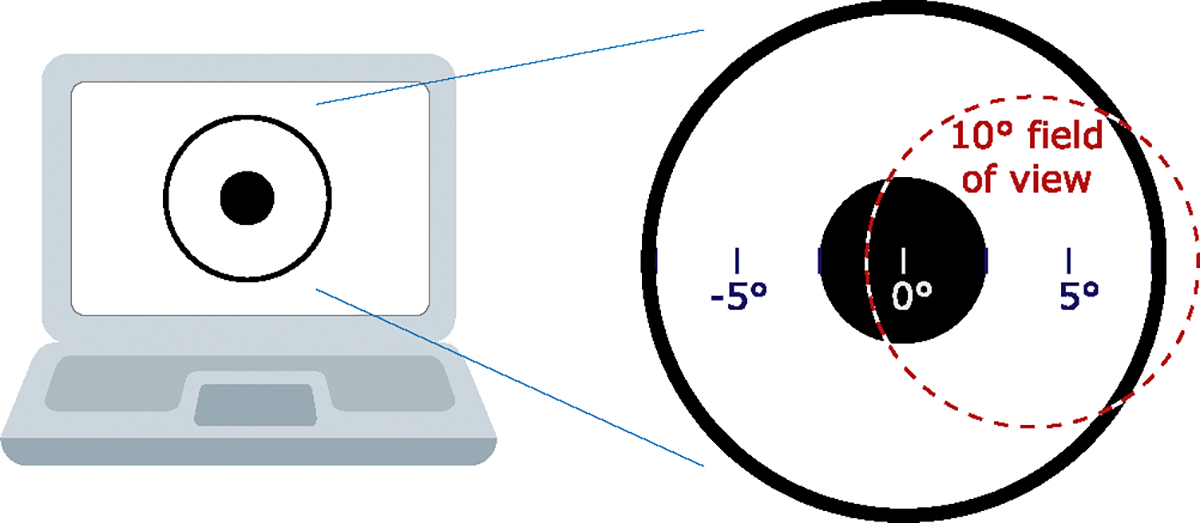
A target-like pattern that would just fail WCAG if it were to flash to white more than 3 times/s, and a person was focused on the center or more peripherally toward the annulus. This figure has a 5°-diameter circle in the middle (with a solid angle area of 0.006 steradians) and an annulus with an inner diameter of 15° and outer diameter of 16.6° (0.012 steradians). Overlaid in a dotted line is a representative, off-center 10° field of view. Laptop illustration from Twitter Twemoji [CC-BY-4.0 license] (https://github.com/twitter/twemoji/ ).

The model of risk derived by Binnie et al. [14] (detailed in the Appendix) can be used to compare the potential risk of simple circular or annular flashing areas. If viewing content on a 19-inch computer display at a preferred distance of 68 cm (26.8 in.) [82], a solid angle of 0.006 steradians is about 1/38 of the display (significantly less than a quarter of the screen). As a lower bound estimate, a 0.006-steradian flashing circle leaves at risk an estimated 9.8% of people who would otherwise respond to the full 19-inch screen flashing. To calculate the upper bound of the risk associated with using the WCAG area threshold, the risk of the target-like figure in Figure 6 was calculated. Using the model to estimate the risk of the flashing figure, 15.8% of people sensitive to full-screen flashing would be likely to respond. Thus, the WCAG criterion (which leaves approximately 15.8% of sensitive people at risk) is much more conservative than the other guidelines that use the 25%-of-the-screen metric for typical computer viewing distances (which would leave approximately 57% of sensitive people at risk). The WCAG criterion also does not require such a stringent threshold as would be true if a 0.006-steradian combined area (created by accumulating all flashing areas from the entire screen) was used (recall that this would leave approximately 9.8% of sensitive people at risk). Note that these estimates of people affected are for maximal luminance flashes at maximally stimulating frequencies, thus the actual risk is significantly lower where the other thresholds are met.

#### 6.2.2 Risk of Mobile.

As shown in Table 2, smart phones are not much riskier than television viewing because of their relatively narrow width in the hand and typical viewing distance. Even if held for close reading at the closest distance measured in [9], smart phone screen viewing angles are comparable to those of computer displays.

#### 6.2.3 Risk of VR and AR.

VR and AR headset viewing covers a very wide angle of view and could be particularly problematic with flashing content. A study investigating the appropriateness of VR for IPS found that VR systems can indeed elicit PPRs similar to standard IPS procedures [66]. However, there are no specific case reports of VR systems provoking PSE in the medical literature [98]. Besides the already-identified PSE risk factors (frequency, area, and changes in luminance or color), there do not seem to be any special additional considerations for VR systems with regards to PSE [32, 98]. Since flashing of 25% of the VR field of view is a very large area (far exceeding 0.006 steradians) and likely to be hazardous, using the WCAG area threshold is recommended.

## 6.3 High-Resolution Displays

Many displays available today have higher resolutions than were available in the past. With higher resolutions, physical pixels are smaller and images can be made clearer. Some devices have such high resolutions that creators usually specify content with logical pixels (such as CSS pixels) that are composed of multiple, smaller physical pixels. Since the guidelines are all in relative units (percent of screen, viewing angle, and solid angle), area thresholds can be transformed to the threshold number of pixels involved. Most guidelines have a 25%-of-the-screen threshold, which is a straightforward calculation. Where content is not expected to be full screen, or where screen viewing angles are significantly different from 10° (such as with computer screen viewing and VR systems), the calculation must be made with some additional assumptions.

### 6.3.1 Content Displayed at Various Sizes.

The WCAG SC is the only current guideline that foresees analyses of content at different sizes and thus needs additional information about the expected viewing environment (screen size + expected viewing distance, expected viewing angle of the screen, etc.). This is given in a WCAG note, where a 341 × 256 pixel rectangle area on a 1,024 × 768 pixel 15- to 17-inch screen was deemed to, “provide a good estimate of a 10° visual field for standard screen sizes and viewing distances” [106]. This corresponds to screens with resolutions of approximately 75–85 **pixels per inch (ppi)**. However, in the CSS 3 specification [104], a CSS pixel is defined as 1/96 of an inch (for print media) or in relation to a reference pixel with a visual angle of 0.0213° (nominally 96 ppi). As older screens are retired, it may be appropriate in the future to move toward a viewing environment defined in CSS pixels, since CSS pixels are technology independent and scale with typical viewing distances (although manufacturers do not always follow the scaling that closely). A 10° viewing angle is approximately 470 CSS px diameter. A square with side lengths of 416 CSS px has a similar area to a 10° circle and could potentially be used for more convenient analysis. Since CSS pixels are also smaller than the pixels defined in the WCAG 2.x series note, there is no increased risk.

Especially with content intended for web use, some content may be viewed at different sizes (e.g., as a thumbnail, a small advertisement, a video embedded in a page, and video played at full screen). Such content should be analyzed for display at the largest expected size. If a specific target device is known—for example, video that only plays on specific kiosks—the specific threshold area in physical or pixel units can be calculated from the guidelines.

#### 6.3.2 Effects of Very Fine Flashing.

**Fig. 7.**
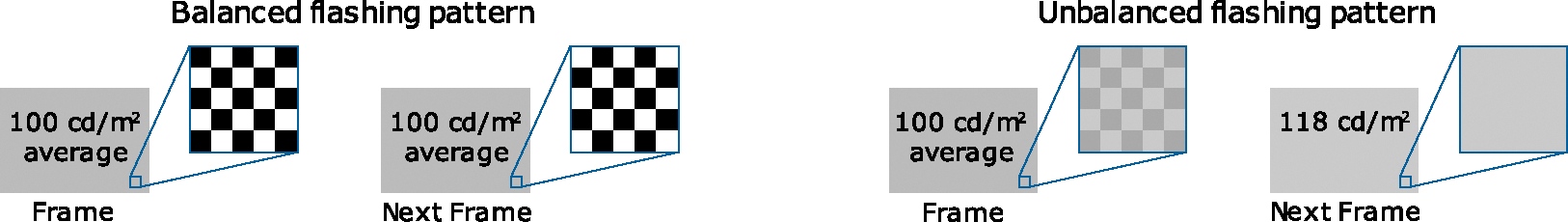
Illustration of balanced and unbalanced flashing patterns. The balanced pattern here is a black-and-white checkerboard that reverses black and white blocks with each frame. Balanced flashing patterns (and white noise) with small enough blocks pass WCAG because of an exception. The unbalanced pattern here shows a first frame made up of a gray checkerboard with brighter blocks are nearly 40 cd/m^2^ brighter than the darker blocks. The second frame is solid gray, which is the same gray as the light gray color of the first frame’s checkerboard. The dark squares are therefore flashing on and off and cover 50% of the screen with a luminance difference exceeding the 20 cd/m^2^ threshold. When viewed at enough distance, the difference in the average luminance is less than the threshold. However, this unbalanced pattern would not pass WCAG.

Many displays are available today where people cannot visually make out individual physical pixels at typical viewing distance (physical pixels smaller than 0.5 arcmin [31]). With such high resolution, what might look like a gray field could instead be made up of a checkerboard or some other fine-grained pattern (see Figure 7). If part of the pattern were to flash, there is a possibility that a pixel-by-pixel analysis might fail while the flashing taken as a whole would not appear as bright visually. Only the WCAG SC has a reference to fine patterns: an exception for white noise or balanced, alternating patterns with blocks of 0.1° (6 arcmin) or smaller. This exception was included in WCAG because there have been no recorded responses to white noise or static on screens at typical viewing distances. No experiments have been done with flashing of patterns that are near the resolution limit of the eye.

This potential problem, of very fine-grained flashing is mostly an academic one. The human visual system is much less sensitive to contrast at high spatial frequencies (i.e., small features), which is primarily due to limits of the eye’s optics [31]. Such patterns do not occur in natural images [108]. White noise and static are balanced and are not provocative. Other very fine-grained patterns are unlikely to be used in practice because such invisible-to-the-eye detail is likely to be lost when video files are compressed.

### 6.4 Faster Frame rates

Displays and content can have faster frame rates than in the past. High frame rates have many potential benefits including increased realism, smoother motion, and an increase in perceived quality [63]. Games are leading the increases in frame rate because it reduces motion blurring and other visual artifacts. However, people are familiar with lower frame rates for both TV and film. Content for TV is produced mainly at 24–60 fps. Most movies are produced for 24 fps, because it has the familiar look of film storytelling. Directors have been experimenting with higher frame rates (notably “The Hobbit: An Unexpected Journey” in 2012 at 48 fps, “Avatar 2” in 2022 with variable framerates of 24 and 48 fps, and “Gemini Man” in 2019 at 120 fps), and Douglas Trumbull did some experiments starting in the 1970s and found that audience physiological stimulation increased with frame rates up to 72 fps [61]. Blu-ray discs support up to 30 fps, Ultra HD Blu-ray up to 60 fps, and Rec. 2020, and Rec. 2100 both go up to 120 fps [51, 52].

When thresholds were initially set, CRT technology was dominant and most television content used interlaced fields with refresh rates of 50 or 60 fields per second, where each field occupied the full screen but at half the resolution. Many analysis tools do a frame-by-frame or field-by-field comparison, which may be inadequate for content at higher frame rates.

#### 6.4.1 Duration of a Flash Transition.

No guidelines specify the timeframe over which a flash transition may occur. A 20-cd/m^2^ transition that might appear from one frame to the next at a “slow” frame rate, might evolve over multiple frames when captured at a faster frame rate. These multi-frame transitions would not be caught by algorithms that only looked at luminance differences between adjacent frames. However, because there is no clear guidance, algorithms might also err on the side of flagging transitions that are too slow. For example, a very slow transition over 750 ms followed by 3 quick flashes (thus potentially 7 transitions in 1 s) could be interpreted as a potential failure without clearer guidance. However, brightness that slowly increases over 750 ms does not look like a flash.

There is no experimental data specific to PSE available for choosing a flash transition duration. Xenon lamp photostimulators used clinically for evoking PPRs have a flash duration of 10 μs [86]. This is in contrast with the 33 ms that each frame is displayed for content at 30 fps on a screen without inherent flicker.

**Fig. 8.**
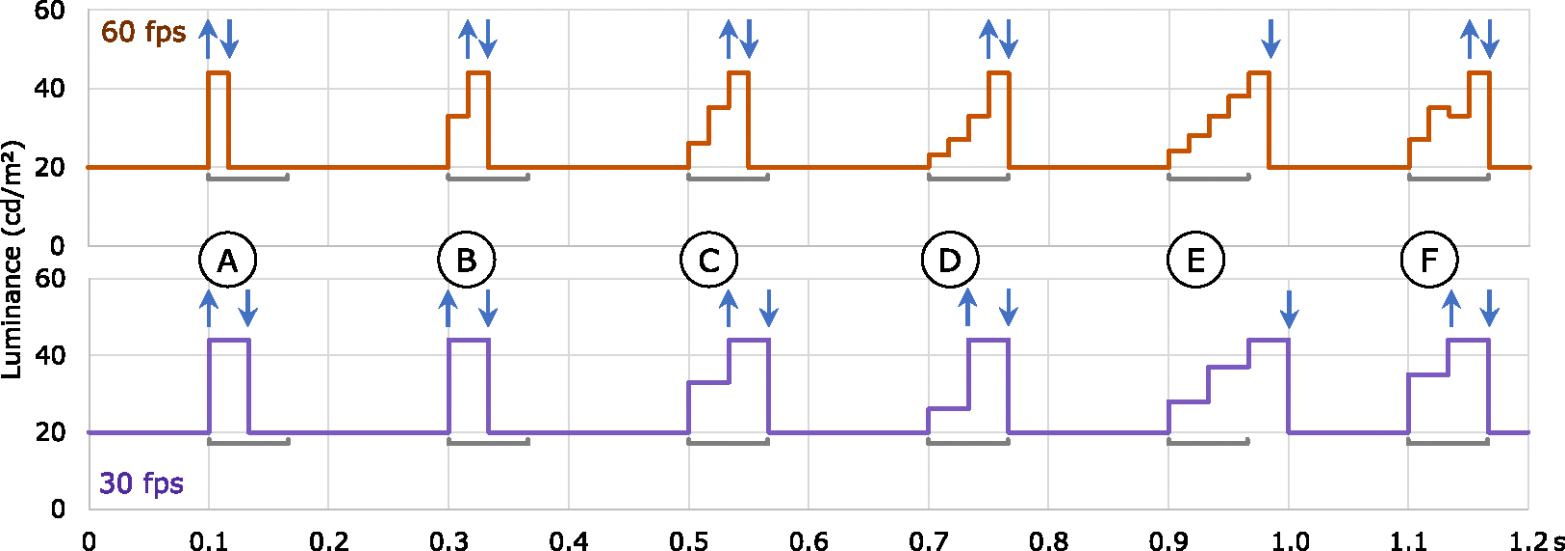
Two waveforms at 60 fps and 30 fps showing transitions that evolve over one or more frames. The 60 ms time windows are represented by the gray lines below each peak. At 60 fps, peaks labeled (A)–(D) take less than 60 ms to exceed the luminance threshold over 1–4 frames, respectively. In (E), it takes longer than 60 ms to exceed the luminance threshold (5 frames at 60 fps), thus there is no up transition. In (F), the waveform is not monotonically increasing, but the luminance threshold is exceeded in less than 60 ms, thus it counts as an up transition over the 4 frames. At 30 fps, a potentially hazardous transition can take 1 or 2 frames (which takes less than 60 ms) to exceed the threshold.

As an initial working value, we propose a value of 66 ms over which a potentially hazardous transition may evolve (see Figure 8). Thus, at common frame rates of 24, 25, and 30 fps, a transition may take place over one or two frames. This is congruent with sample videos that we have tested using the PEAT system, where two-frame transitions fail and three-frame transitions do not. With a 66 ms time window, transitions may take up to 4 frames for 50 fps and 60 fps content, up to 8 frames at 120 fps, and so on.

#### 6.4.2 Synchronicity of Flashing Areas.

With most technologies, the pixels of a display are not all changed at the exact same instant (many types of screens are updated row by row), so the pixels of a flashing area within a single video frame are already not strictly synchronized. With faster frame rates, a flash transition may also roll spatially over an area and may be slightly out of sync. For example, 15% of the screen may transition in one frame and a contiguous additional 15% might also transition in the next frame 8 ms later (at a fast 120 fps). How close in time do transitions of pixels or areas of the screen have to be in order for their areas to be considered part of the same flash transition? There is no experimental data available for choosing a time window of synchronization specifically for PSE. Photostimulators used for diagnosis of PSE flash the entire area at the same instant with a very short burst of light. As frame rates for movies, games, and VR increase, combining rapidly occurring contiguous frame transitions in the same direction might be appropriate. However, it would also make it more intensive to calculate which transitions are associated, since individual pixels could undergo multiple transition within several frames.

**Fig. 9.**
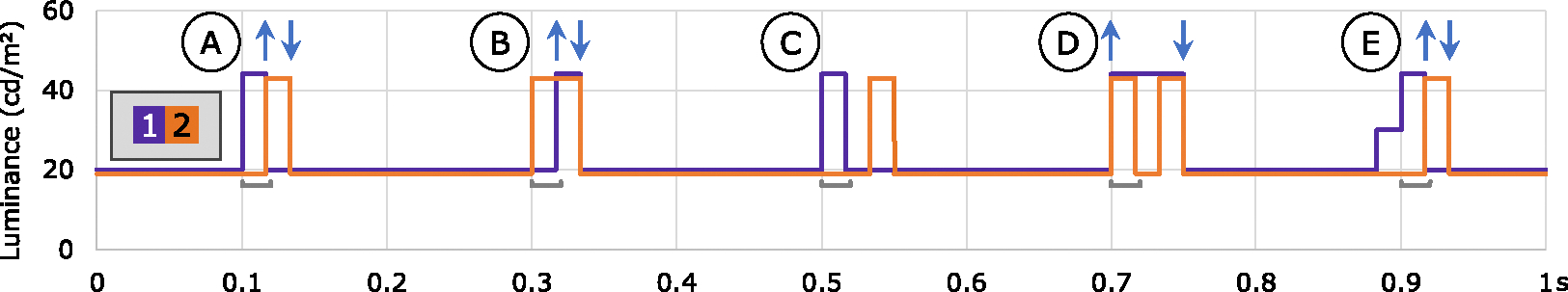
Luminance waveforms at 60 fps for two areas of the screen that exceed the area threshold in sum, but not separately. The synchronicity time window of 20 ms is represented by the gray lines below the waveforms. In (A) and (B), the leading edges of the two areas are within 20 ms, thus the areas are summed. In (C), the leading edges are more than 20 ms apart, thus are not summed. (D) shows Area #2 flashing twice within a single flash of Area #1, which counts as two transitions where the areas transition in the same direction simultaneously. (E) shows Area #1 undergoing a two-frame luminance ramp that exceeds the luminance difference threshold of 20 cd/m^2^ within 20 ms of the leading edge of Area #2, thus the areas are summed.

A low estimate of CFF can guide a working value for synchronicity. As an initial working value, we suggest a synchronicity time window of 20 ms, which corresponds to a conservative CFF of 50 Hz. This would mean that at frame rates from 50 to 99 fps, a flashing area that crosses two frames would count as a single transition (see Figure 9). This recommendation encompasses two fields (occurring at either 50 or 60 fields per second) of interlaced analog television of the past. For the many movies and TV shows today that have frame rates in the 24–30 fps range, synchronicity would still be analyzed frame-by-frame as in the past. Nearly-synchronized areas that are to be considered together for analysis can only occur at higher frame rates.

#### 6.4.3 Maximum Rate of Flashing.

The risk of PPR is low with fast flashing over 65 Hz [42]. Only the ISO standard specifies 65 flashes or more in a second as safe, but there remains a potential risk with the way the provision is worded. With very fast displays within a single second of time, it would be possible to have a period of flashing at say 50 Hz followed by flashing at a faster rate such that there were a total of 65 flashes or more. Flashing at 50 Hz has the potential of triggering PPRs for 49% of those who are photosensitive [42].

To prevent this potential mixed-rate flashing, the safe upper limit of flashing might be worded:

“Fast flashes, where leading edges are separated by 15 ms or less, can be merged and counted as a single flash, since a flash rate of 65 Hz or faster is associated with significantly reduced risk.”

Displays and computers are now becoming fast enough to display content faster than 130 fps (which is the minimum frame rate that could display 65-Hz flashing content). While such content is rare, for futureproofing and harmonization, other guidelines should consider adopting language similar to that above to include 65 Hz or faster as safe levels of flashing.

### 6.5 Other Aspects for Research and Harmonization

#### 6.5.1 Differing Thresholds between Japan and Other Guidelines.

Four of the guidelines have similar thresholds that apply evenly to various content, from flickering areas to fast cuts and scene changes (see Table 3). Compare this to the PSE thresholds in Japan, which are more complex. The Japan guidelines have different units between SDR and HDR content and are further split into ranges, which each have their own flash rate threshold.

In the Japan guidelines, there are essentially three ranges of intensity, which will be labeled here for convenience as “moderate flashing,” “intermediate flashing,” and “scene changes.” Each of these ranges applies to three types of content: (1) SDR content, (2) HDR content where the darker state is *<*160 cd/m^2^, and (3) brighter HDR content where the darker state is ≥160 cd/m^2^ (Table 4). *Moderate flashing* is allowed at a rate of 5 flashes/s for up to 2 s—this is less conservative than the other guidelines which cap all flashing at 3 flashes/s. There is no data specifically measured at moderate brightness levels, but with a bright photostimulator at 5 flashes/s, 11% of patients responded (compared to 3% responding at 3 flashes/s of other guidelines) [42]. The actual risk of a response is expected to be lower at these moderate levels of brightness. *Scene changes* involve most of the screen at an intermediate or greater level of flashing. Scene changes are limited to only 3 per second—only 2% of patients are expected to respond to a photostimulator flashing at a rate of 1.5 Hz [42]. This is more conservative than the other guidelines. *Intermediate flashes* in the Japan guidelines are similar to other guidelines with the same limit of 3 flashes per second.

Harmonizing on a single standard is not clear in this case. With scene changes, inversions, fast cuts, and very large areas of flashing, the Japan guidelines are more conservative than the others. Conversely, in limited cases with moderate flashing, the Japan guidelines give creators more creative flexibility than would be allowed under the other guidelines. Developers of analysis tools and creators who might broadcast television content in Japan need to be aware of these differences between guidelines. Tools that are going to be used internationally will need special analyses to support broadcast content in Japan.

#### 6.5.2 Pacing of Flashes.

Except for ITU-R and Ofcom, most guidelines do not reference the pacing of flashes and only prohibit more than three flashes in one second. However, in the case of ITU-R and Ofcom when leading edges of flashes are separated by 334 ms or more, then it is considered safe. Thus, a sequence of two quick, bright flashes with a half-second pause followed by two more quick flashes would pass ITU-R and Ofcom, but not the other guidelines. The foundational experiments [42] and current clinical practice [57] use flashing at constant rates, thus there is no specific data to support or contradict the leading-edge-to-leading-edge qualification of ITU-R or Ofcom.

Because it is more conservative, we suggest using the “no more than 3 flashes in any second” threshold (without qualification) that most guidelines use. Note however, that analyses according to ITU-R and Ofcom thresholds might be more memory efficient since a smaller buffer of frames (334 vs. 1,000 ms) needs to be analyzed.

#### 6.5.3 Red Flash Color Difference.

Most of the guidelines do not specify details about what constitutes hazardous red flashes, just that flashing involving red is particularly hazardous. The two that have specific thresholds (ISO and WCAG 2.2) are now harmonized, but the earlier versions of WCAG differ significantly in the required difference in color between the saturated red and other states. To date, most experiments on the effects of flashing alternating colors on PPR have used colors that differ greatly in hue [12, 75, 96, 113], thus there is limited information for picking a color difference threshold. More photosensitive responses have been found with red-blue flashing than with red-green flashing or red-off flashing [75]. More research is needed to understand the relationship between PSE and chromatic flashing to set better potential hazard thresholds.

The earlier WCAG 2.0/2.1 threshold between the two opposing color states of a red flash is more conservative than the harmonized ISO and WCAG 2.2 threshold (refer back to Figure 2). The earlier WCAG 2.0/2.1 threshold is 3D in nature (using the three sRGB primaries). Chromaticity diagrams, as with the ISO and WCAG 2.2 threshold, are 2D and thus are a flattening of a color space. It is thus possible that some colors with the same chromaticity coordinates (thus not a transition in ISO) might differ enough in WCAG 2.0/2.1 critical values.

As input to the WCAG 2.2 working group in 2022, the authors suggested harmonizing on the ISO difference between colors (*>*0.2 units on the 1976 CIE UCS chromaticity diagram). Such colors are more visually distinct compared to the borderline cases that might be caught under the WCAG criterion. Under the earlier WCAG 2.0/2.1 thresholds, both states of a red flash could be saturated red (e.g., #FF0000 and #FF3636).

#### 6.5.4 Saturated Red Definition.

The exact source for the saturated red equation (Equation (2)) in ISO and WCAG is unclear, but it may have initially been a working definition from the HardingFPA tool and Cambridge Research Systems, which consulted on the relevant WCAG SC. Cambridge Research Systems reports Equation (2) as their own definition for saturated red “in the absence of a specific definition” [45]. They further report that the red flash analysis excludes colors, “which may be perceived as orange or purple” [45]. However, borderline colors that qualify as “saturated red” under ISO, WCAG, and HardingFPA include some colors that many might not characterize as red (see Table 5).

There is little research available for setting a specific definition for saturated red. Red does not have to be pure red to be provocative [45]. In early studies, there was disagreement on whether red flashes were a hazard or not, but this discrepancy was due to experiments using red colors of different wavelengths (peak 580 nm compared to wavelengths beyond 600 nm) [41].

There is also an open question about dark, low-luminance red colors (i.e., deep shades of red). It would seem reasonable that hazardous red colors would need to both (1) be within a range of red hues and (2) have a certain minimum luminance. The red threshold used in ISO and WCAG 2.2 applies to bright red and to red colors that approach black. In the older WCAG 2.0 and 2.1, there was effectively a floor (one color of which is #470000) because dark red flashing against black had to meet a critical difference of at least 20 units between values calculated with Equation (3).

Without additional research, the saturated red threshold in current standards is the best available. It is likely to be conservative, particularly with flashing that involves darker shades of red.

#### 6.5.5 Thresholds for Failures over an Extended Time Period.

Continued flashing can have a cumulative effect for some people. Flashing that might not initially cause a response may cause a response with extended viewing. Three standards (ISO, Ofcom, and ITU-R) mention extended flashing over 5 s or more, but none give any thresholds. In the HardingFPA tool, an extended failure is raised when, “luminance or red flashing occurs close to the guideline limit … in more than 80% of video frames in the last 5 s” [45] but guidance on specific thresholds is not published. Common clinical practice for photic stimulation is to present separate 5-s trains of flashes at different flash rates with eyes open and closed [57], but none of the testing is done while varying the luminance or area of flashing.

In light of this, developers who are interested in building tools that can give extended failures should use variable parameters for luminance and area.

#### 6.5.6 Effects of Movement.

In the human visual system, motion is perceived differently than flicker. Compared to static and oscillating patterns, patterns that drift slowly in one direction result in significantly less synchronized excitation of neurons [16]. The ISO and Ofcom guidelines give specific PSE guidance for patterns and state that potentially hazardous patterns are exempt if they exhibit smooth flowing motion “across, into, or out of the screen in one direction” [49, 69]. In Wilkins, Emmett, and Harding [109], drifting patterns are allowed to have a larger number of light-dark pairs of stripes compared to stationary patterns or patterns that change direction, oscillate, flash, or reverse. In evaluating various videos for potential PSE risk, Binnie et al. listed examples of motion that might give spurious results if not otherwise accounted for: “running feet, trains passing, shaking of a coin box, fast pans, etc.” [14]. An example analysis framework given in ITU-R BT.1702 includes motion estimation and motion compensation steps before PSE analysis in order to “reduce spurious detection due to panning and object motion” [50]. Documentation for the HardingFPA system [22] also states that motion and noise compensation is performed before flash analysis.

Unfortunately, there is no specific, actionable guidance available on motion compensation thresholds that are appropriate for analysis. When is motion so fast that it is perceived as a flicker rather than motion? Are there circumstances will the eyes track a moving and flashing/pulsing object such that there is a risk of a photosensitive response, even if the object moves outside of the initial 10° field of view?

## 7 Proposed Updates to PSE Guidance

With the changes of technology and opportunities for harmonization, the PSE guidance that is currently available could be updated. With increasing interest by developers building tools, the guidance needs to be clearer and cover where technology is today and in the near future.

The following proposed guidance includes new provisions on the duration and synchronicity of flash transitions (Tables 6 and 7). These new provisions are marked here as “working values” that should be considered as parameters to include in new tools and that members of standards bodies should also consider when updating thresholds and guidance. The language may be adapted to the style of different guidelines.

The proposed provision and definitions are available for potential reuse at https://github.com/traceRERC/pseGuidelines/

## 8 Future PSE Tools

Analysis and screening tools must also adapt as technology advances. Many of today’s PSE tools are designed for screening and checking content that has already been created, such as animations, movies, and videos. Creators of games and virtual spaces, however, cannot foresee all of the ways that a person could interact with and view their content. For example, people might experience a flashing effect by quickly changing their viewpoints back and forth or moving in close proximity to illuminated or reflective virtual objects and surfaces. With video games and as the use of VR increases, it will become more important to be able to assess the risk of content that is being generated in real-time when it is viewed.

With generated content, modifying the video stream in real-time has the potential to be helpful, at least to those who are aware of their photosensitivity. Very restrictive approaches, such as content blocking or blanking the screen may be fine in some circumstances, but may be unacceptable to people who want to interact in a virtual space or play games. In Japan, there was some work on adaptive temporal filters to reduce frame-to-frame flicker over 10 Hz [67], which was shown to eliminate PPRs in the 11 photosensitive patients that were tested [68]. This filtering was extended to filtering geometric pattern flicker [90]. The FFmpeg vf_photosensitivity.c filter [74] can make real-time changes to video, which look somewhat like multi-frame cross-fading with large luminance changes. With Video Flashing Reduction, the developers have proposed reducing contrast as a mitigation strategy [7]. Neither of these more recent mitigation strategies have been formally tested yet with people with photosensitivity. There are many other possible schemes for muting the effects of flashing, including limiting changes in luminance, freezing or cross-fading frames, desaturating the red channel, and other strategies to reduce the potential to provoke a response. Furthermore, modifications and filters can be applied at different points: filters might be applied to a whole video known to contain a hazardous sequence, filters might be applied whenever a single, large transition occurs or after a couple flashes that might form the beginning of a hazardous sequence, or filters might be applied in a graduated manner to match how provocative the source video may be. More research is needed in this area to see what strategies are effective and acceptable to different people under different circumstances.

More research also needs to be done with newer display technologies besides televisions and other flat displays. Close viewing and more immersive displays could increase risk because more of the brain would be stimulated. A novel color stimulator with light emitting diodes arranged to curve 180° around participants’ faces was used to study PPRs to chromaticity changes [75]. One team is creating a system (VR-Photosense) using VR headsets and machine learning for performing IPS with patients [66]. In an interview study with five people with photosensitivity, researchers found that people were wary of VR and their ability to keep themselves safe in a VR environment, but also saw potential future benefits with VR environment customization, better warnings (automated or crowdsourced), and with shared viewing and control with trusted family and friends [85]. Hazard screening and mitigation will need to be developed for VR and AR because it is more immersive than television and computer viewing.

## 9 Conclusion

There are five major standards and guidelines for PSE for different technology domains. These guidelines are not fully harmonized and can be difficult or confusing to understand. Furthermore, the guidelines may need updates and more primary research to account for risks that may be associated with new technologies and content formats. Future guidelines should incorporate newly identified potential risk factors. Since the only practical way to apply these guidelines is with automated tools, the goal is that developers and others will be able to use PSE guidelines to create tools that can be used in creative workflows, for pre-screening content before broadcast or dissemination, and to actively mitigate potentially hazardous content.

## Figures and Tables

**Table 1.** Summary of Flashing Guidance

	Expert consensus [44]	ITU-R [50]	Ofcom (as ITC before 2003) [69]	NHK/JBA (moderate contrast) [55]	NHK/JBA (higher contrast) [55]	ISO [49]	WCAG 2.× SC 2.3.1 [106]
Dates updated	2005	2005, 2018, 2019, and 2023	1994, 1999, 2001, 2002, 2005^a^, and 2017^a^	1998, 2006, and 2020	1998, 2006, and 2020	2016, 2021^a^	2008, 2018^a^, and 2023
Flashes in a time frame	>3 flashes within 1 s	>3 flashes within 1 s unless leading edges are separated by ≥334 ms	>3 flashes in 1 s unless leading edges are separated by ≥9 frames	>5 flashes/ s with a maximum 2-s window	>3 flashes within 1 s	>3 flashes within 1 s	>3 flashes within 1 s
Brightness difference between states (SDR)	≥20 cd/m^2^	≥20 cd/m^2^ when darker state <160 cd/m^2^	≥20 cd/m^2^ when darker state <160 cd/m^2^	10–20%	>20%	≥20 cd/m^2^ when darker state <160 cd/m^2^	≥0.1 relative luminance when darker state <0.8 relative lum.
Brightness difference between states (HDR)	-	≥20 cd/m^2^ where darker state <160 cd/m^2^ and > 1/17 Michelson contrast otherwise	-	20–40 cd/m^2^ where darker state <160 cd/m^2^ and between 1/8 and 1/4 brightness change otherwise	≥40 cd/m^2^ where darker state <160 cd/m^2^ and >1/4 brightness change otherwise	-	-
Red flash transition	To or from a saturated red	To or from a saturated red	To or from a saturated red	Handle flashes of pure red with particular care	Handle flashes of pure red with particular care	Saturated red and opposing color change given in standard and annex	Saturated red and opposing color change given in notes that differ between 2.0/2.1 and 2.2
Area of flashing	>0.006 steradians (> 25% of central 10° of visual field)	>25% of full screen	>25% of displayed screen area	>25% of the screen	>25% of the screen	>25% of displayed screen area	>0.006 steradians within any 10° field of view (>25% of any 10° field of view)
Inversions or scene changes	-	Analyzed as flashing	Analyzed as flashing	N/A	>3 such transitions in 1 s	Analyzed as flashing	-

a Reaffirmed or republished with no substantive changes to PSE guidance.

This table is a summary of the guidelines. Please see the original guidelines for conformance purposes.

**Table 2.** Viewing Angles and Risks of Various Screens

	Full screen viewing angles (Long × Short dimensions)	Percentage of screen forming a solid angle of 0.006 steradians	Estimated response rate of people with PSE to flashing at maximally stimulating rate and modulation on a contiguous screen area of 25%^a^

20-in. TV (4:3) viewed at 7*H*^b^ [14]	10.9° × 8.2°	21.2%	12%
60-in. TV (4:3) viewed at 5*H*^b^ [14]	15.2° × 11.4°	10.9%	23%
65-in. TV (16:9) viewed at 3.2*H*^b^ reference viewing distance [52]	31.0° × 17.8°	4.5%	42%

6.1-in. smart phone (19.5:9) viewed at 32 cm (12.6 in.) average distance [9]	24.8° × 11.6°	10.5%	23%
6.1-in. smart phone (19.5:9) viewed at 19 cm (7.5 in.) close distance [9]	40.6° × 19.4°	3.8%	47%

10.1-in. tablet (16:10) viewed by children at 36.8 cm (14.5 in.) average distance [79]	32.9° × 20.9°	3.2%	51%
10.1-in. tablet (16:10) viewed by children at 29 cm (11.4 in.) close distance [79]	41.1° × 26.4°	2.0%	65%

15-in. computer display (4:3) viewed at 56 cm (22.0 in.) [106]	30.5° × 23.1°	2.7%	63%
1,920 × 1,080 CSS reference pixel (1/96 dpi) display (16.9) at 71.1 cm (28 in.) [104]	39.3° × 22.7°	2.7%	56%
19-in. computer display (4:3) viewed at 68.0 cm (26.8 in.) [82]	31.0° × 24.0°	2.6%	57%
27.5-in. computer display (16:9) viewed at 75.7 cm (29.8 in.) [82]	42.7° × 27.5°	1.9%	67%

VR headset^c^	90° × 90°	0.3%	100%
VR headset with a wide field of view^c^	174° × 114°	0.2%	100%

a This calculation is done with model in [14] using a visual angle of 4/3rds of the short screen dimension for the full-screen normalization. See the Appendix for equations.

b *H* is the height of the screen.

c VR headset fields of view from crowdsourced database available at https://www.infinite.cz/projects/HMD-tester-virtual-reality-headset-database-utility

**Table 3.** Summary of Flash Thresholds from ISO, ITU, Ofcom, and W3C

Content type	Darker state	Content	Magnitude of change	Screen area	Flash rate
SDR —W3C [106]	Where darker state is <0.8 relative luminance [<160 cd/m^2^]^a^	Flashing	≥0.1 relative luminance [≥20 cd/m^2^]	0.006 steradians of any 10° field of view	>3 flashes/s
SDR —ISO [49] —ITU-R [50] —Ofcom [69]	Where darker state is <160 cd/m^2^	Flashing or scene changes	≥20 cd/m^2^	25–100% screen area	>3 flashes/s
HDR —ITU-RITU	Where darker state is ≥160 cd/m^2^	Flashing or scene changes	>1/17 Michelson contrast	25–100% screen area	>3 flashes/s

a Values in square brackets “[]” are calculated assuming a maximum screen luminance of 200 cd/m^2^.

**Table 4.** Summary of Flash Thresholds from NHK/JBA [55]

Content type	Darker state	Intensity range^a^	Magnitude of change	Screen area	Flash rate
SDR	-	Moderate flashing	10–20% brightness (IRE)	25–100%	>5 flashes/s for 0–2 sec duration AND >3 flashes/s after 2 sec
		Intermediate flashing	20–100% brightness (IRE)	25–80%^b^	>3 flashes/sec
		Scene changes	20–100% brightness (IRE)	80–100%	>1.5 flashes/sec
HDR	Where darker state is <160 cd/m^2^	Moderate flashing	20–40 cd/m^2^	25–100%	>5 flashes/s for 0–2 s duration AND >3 flashes/s after 2 s
		Intermediate flashing	>40 cd/m^2^	25–80%	>3 flashes/s
		Scene changes	>40 cd/m^2^	80–100%	>1.5 flashes/s
	Where darker state is ≥160 cd/m^2^	Moderate flashing	1/8–1/4 brightness change from dark state [Michelson contrast 1/17 –1/9]^c^	25–100%	>5 flashes/s for 0–2 s duration AND >3 flashes/s after 2 s
		Intermediate flashing	>1/4 brightness change from dark state [Michelson contrast > 1/9]	25–80%	>3 flashes/s
		Scene changes	>1/4 brightness change from dark state [Michelson contrast >1/9]	80–100%	>1.5 flashes/s

a Author categorization labels are for convenience.

b The 80% of screen area threshold for scene changes comes from [22].

c Michelson contrast values marked in square brackets “[]” were calculated using Equation (1).

**Table 5.** Select Colors That All Qualify as Saturated Red (ISO [49], WCAG [106], and HardingFPA [45])

Color value (sRGB hex)	Author’s color description^a^	Color Swatch	Chromaticity coordinates (u’, v’)	Equation (2) value (≥0.8 is sat. red)	Equation (3) value (WCAG 2.0 and 2.1)

#FF0000	Red		0.4507, 0.5229	1	320
#FF8800	Reddish orange		0.3091, 0.5401	0.8024	241.22
#FF0088	Reddish magenta		0.3910, 0.4438	0.8024	241.22
#FF6363	Reddish pink		0.3425, 0.4995	0.8003	240.14
#470000	Extremely dark red		0.4507, 0.5229	1	20.16
#080101	Nearly black		0.3423, 0.4995	0.8000	0.5828

a Author-provided color description is for accessibility.

**Table 6.** Proposed Provision

There are no 1-s time spans, where there are more than 6 luminance transition or red transition counts where the transitions meet all the following criteria:
— Alternate in direction,
— Equal or exceed the critical transition difference,
— Are of qualifying duration, and
— Have a summed area (including adjoining or nearby areas that are sufficiently synchronized to be treated as the same transition) that is equal to or greater than the potentially hazardous area threshold.

Underlined terms are terms with proposed definitions.

**Table 7.** Proposed Definitions

Term	Definition	Reference

Counts (transition counts)	Number of luminance transitions or red transitions that are not parts of fast flashes. Note: Luminance transitions and red transitions are counted separately. Note: It is possible for a single transition to count as both a luminance and red transition. Note: Fast flashes are counted differently (see below).	ISO, ITU, NHK/JBA, Ofcom, and WCAG

Luminance transition	Transition between higher and lower luminance states (in either direction).	ISO, ITU, NHK/JBA, Ofcom, and WCAG
Red transition	Transition between a saturated red state and a different color state where the difference between states equals or exceeds the critical transition difference (in either direction, regardless of luminance). Note: The color states in a sequence of flashes do not need to match each other.	ISO, ITU, NHK/JBA, Ofcom, and WCAG

Fast flashes	Sequence of flashes where every second transition (i.e., every transition in the same direction) is separated by 15 ms or less.	Section 6.4.3
	—When calculating transition counts, all transitions in the same direction in the fast flash sequence should be merged and counted as one transition (thus the sequence of fast flashes is counted as two transitions total). This merged flash should be analyzed (separately) as occurring at the start and again at the end of the sequence of fast flashes (but not both in the same analysis).	
	Note: A flash rate of 65 Hz or faster (where leading edges of flashes are spaced 15 ms or less) is associated with significantly reduced risk.	

Critical transition difference	For a red transition, —a color difference that is 0.2 units or greater on the 1976 CIE UCS chromaticity diagram between a saturated red state and a different color state.	*Red*: ISO, WCAG2.2, Section 6.5.3
	For a luminance transition, —a difference between a lower and higher level of luminance (going in either direction) of —if the lower luminance state is < 80% of the reference luminance (see notes): —10% of the reference luminance, or —else if the lower luminance state is ≥80% of the reference luminance: —a Michelson contrast between states of 1/17.	*Luminance*: ISO, ITU, NHK/JBA, Ofcom, WCAG
	Note: Reference luminance differs across technologies:	
	—For sRGB: Both the peak and reference luminance for evaluation are a relative luminance of 1.0. The relative luminance (Y in the CIE XYZ color space, which ranges from 0 to 1) can be calculated with the transfer function specified in IEC 61966–2-1.	
	—For SDR television with standard color range (ITU-R BT.709) or wide color range (ITU-R BT.2020): use the electro-optical transfer function in Annex 1 of ITU-R BT.1886 with both a reference luminance and screen luminance for white (*L_W_*) of 200 cd/m^2^ and screen luminance for black (*L_B_*) of 0.0 cd/m^2^.	
	—For PQ HDR content: use the reference luminance of 200 cd/m^2^ and transfer functions specified in PQ tables in ITU-R BT.2100.	
	—For HLG HDR content: use the reference luminance of 200 cd/m^2^ and transfer functions specified in HLG tables in ITU-R BT.2100.	
	—For content to be displayed on known display hardware: use the reference white luminance value for the display, which is the peak luminance for SDR content. If the viewing environment is known, the reference luminance may be for vision adapted to that environment.	
	Note: For typical indoor viewing environments, a reference luminance of 200 cd/m^2^ is appropriate, which leads to a critical transition difference of 20 cd/m^2^ when the darker state is < 160 cd/m^2^. Note: Theater viewing is often done with dark adapted viewing and a peak/reference luminance of 48 cd/m^2^, which leads to a critical transition difference of 4.8 cd/m^2^ when the darker state is < 38.4 cd/m^2^.	
	Note: For HDR content, it is assumed that the content has been mastered so that 200 cd/m^2^ is the reference white and that greater luminance is generally reserved for reflections, shine, and specular highlights of relatively small areas.	
	Note: Luma, often denoted Y’, is not the same as relative luminance, which is often denoted Y. Calculating relative luminance Y accurately from luma also requires two other components (for example, the C_B_ and C_R_ components of the Y’C_B_C_R_ color space).	

Qualifying duration (working value)	The time over which a single transition may take place of 66 ms or less. Note: A slow change in luminance or color that takes more than 66 ms to reach or exceed the critical transition difference does not count as a flash transition. Note: This is a working value that may change as more is learned about PSE.	Section 6.4.1

Potentially hazardous area threshold	An area or collection of areas where the combined area equals or exceeds: —For content on a known screen size and typical viewing distance: —0.006 steradians of any 10° field of view (equivalent to 25% of any 10° field of view) at the typical viewing distance. —For content only shown on televisions of unknown screen sizes: —25% of the screen.	ISO, ITU, NHK/JBA, Ofcom, WCAG, Section 6.2.1, Section 6.3.1
	—For content displayed on screens of unknown type and size:	
	—25% of any 416 × 416 px subarea of the content (where the “px” unit is a CSS pixel that is defined as 0.0213° viewing angle), analyzed when content is displayed at its largest size (without the user applying additional zoom).	
	Note: A 23-inch (diagonal) Full HD monitor viewed at arm’s length can be used as a reference screen for scaling content displayed on screens of unknown types and sizes. This reference screen is 1,920 × 1,080 px (i.e., 0.0213° CSS pixels) and viewed at 71.1 cm (28 in.).	
	Note: See W3C CSS Values and Units Module Level 3 for definition of reference pixel (px).	
	Note: The 25%-of-the-screen metric for television content was chosen for backwards compatibility. With modern displays and preferred viewing distances, the risk associated with 25% of the screen is increased compared to when the standards were first created.	

Sufficiently synchronized (working value)	When transitions are in the same direction and occur within 20 ms of each other. Note: Areas are added together for analysis when they transition in the same direction within 20 ms of each other. Note: This is a working value that may change as more is learned about PSE and displays with high frame rates.	Section 6.4.2

Michelson contrast (equation)	$\frac{L_{\mathrm{High}}\;-\;L_{\mathrm{Low}}}{L_{\mathrm{High}}\;+\;L_{\mathrm{Low}}}$ where $L_{High}$ and $L_{Low}$ are the luminance of the high and low luminance states, respectively.	ITU

Saturated red (equation)	Colors where the red channel component is 80% or more of the total RGB color signal after gamma correction and any other transformations. $\frac{R}{R+G+B}\geq 0.8$ where *R*, *G*, and *B* are the normalized linear values of the red, green, and blue channels after gamma correction and any other transformations	ISO, WCAG, Section 6.5.4

Underlined terms are terms with proposed definitions.

**Fig. A1.**
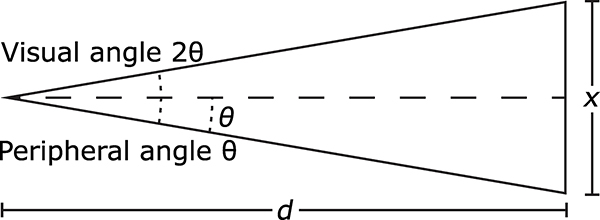
The relationship between visual angle (2θ), peripheral angle (θ), viewing distance (d) and object size (x).

## CCS Concepts:

• Human-centered computing → Accessibility design and evaluation methods; *Accessibility systems and tools*; • General and reference → Metrics; • Software and its engineering → *Software safety*; • Information systems → Multimedia content creation; • Applied computing → Law;

## Appendix

### A Equations for Viewing Angles and Proportion of People Affected

The visual angle, 2θ, can be calculated with the following equation:

$$2\theta =2\;{tan}^{-1}\frac{x}{2d}$$

where d is the viewing distance and x is the physical size of the viewed figure, both in the same units (see Figure A1).

The solid angle (in steradians) of a right circular cone only depends on the angle at the apex, 2θ (expressed in radians).

solidangle=2π(1−cosθ).

A model of risk derived by [14] can be used to compare the potential risk of simple circular or annular flashing areas and was used to generate estimated response rates in Table 2 in the text. The first step is to calculate the percentage of the visual cortex that is stimulated, Q (range 0–100), with the peripheral angle θ (half of the visual angle), which comes from the work of [27]

$$Q=100\left(1-e^{-0.0574\theta }\right).$$

Given the percentage of visual cortex stimulated, the proportion of patients affected by maximal flashing of the circular area is given by

$$\mathrm{Proportion}\;\mathrm{affected}\;(\mathrm{not}\;\mathrm{normalized})\;=\left\{\begin{array}{ll} 1 & \mathrm{if}\;Q>56.37\\ 0.21Q-0.1827 & \mathrm{if}\;8.75<Q<56.37\\ 0 & \mathrm{if}\;Q<8.75 \end{array}.\right.$$

In Table 2, the proportion of patients affected by the full screen was calculated using a circle with a diameter of four-thirds (4/3) the length of the shorter dimension of the rectangular screen.
